# Shrimp Lipid Bioactives with Anti-Inflammatory, Antithrombotic, and Antioxidant Health-Promoting Properties for Cardio-Protection

**DOI:** 10.3390/md22120554

**Published:** 2024-12-11

**Authors:** Paschalis Cholidis, Dimitrios Kranas, Aggeliki Chira, Evangelia Aikaterini Galouni, Theodora Adamantidi, Chryssa Anastasiadou, Alexandros Tsoupras

**Affiliations:** 1 Hephaestus Laboratory, School of Chemistry, Faculty of Sciences, Democritus University of Thrace, Kavala University Campus, St. Lucas, 65404 Kavala, Greece; paoholi@chem.duth.gr (P.C.); dikrana@chem.duth.gr (D.K.); aggelikichira@gmail.com (A.C.); evgalou@chem.ihu.gr (E.A.G.); theadam@chem.duth.gr (T.A.); 2 Fisheries Research Institute, Nea Peramos, 64007 Kavala, Greece

**Keywords:** shrimp, anti-inflammatory, antioxidant, antithrombotic, PUFA, astaxanthin, carotenoids, lipid bioactives, chronic diseases

## Abstract

Marine animals, especially shrimp species, have gained interest in research, due to the fact that they contain a plethora of biomolecules, specifically lipids, which have been proven to possess many health benefits in various diseases linked to chronic inflammation or other exogenous factors. This review refers to the lipid composition of a large number of shrimp species, as well as the effects that can alternate the lipid content of these crustaceans. Emphasis is given to the potent anti-inflammatory, antioxidant, and antithrombotic properties of shrimp bioactives, as well as the effects that these bioactives hold in other diseases, such as cancer, diabetes, neurodegenerative disorders, and more. The various health-promoting effects deriving from the consumption of shrimp lipid bioactives and the usage of products containing shrimp lipid extracts are also addressed in this study, through the exploration of several mechanisms of action and the interference of shrimp lipids in these biochemical pathways. Nevertheless, further research on this cultivatable edible species is needed, due to their existing limitations and future prospects which are discussed in this paper.

## 1. Introduction

Over the years, chronic illnesses such as cardiovascular diseases, diabetes, obesity, several types of cancer, Alzheimer’s, Parkinson’s, and many more tend to be more prevalent even in younger people, due to many factors including the lack of exercise, dietary habits, and modern lifestyle. The World Health Organization (WHO) has reported that cardiovascular diseases are the leading causes of death globally and has pursued numerous approaches, so they can be prevented and treated. Many researches have claimed that chronic inflammation, thrombosis, and oxidative stress are directly connected to this phenomenon [1,2,3]. Chronic inflammation is most commonly caused by infections that cannot be resolved by endogenous resistance, host defense, or other mechanisms, but it may also arise from substances that cannot be degraded, by genetic predisposition, by genetic sensitivities, by prolonged exposure to chemicals, or from pathogenic microorganisms that are also causes of chronic inflammation [4].

Stress is another common factor that is actively linked to cardiovascular diseases due to its effects on the volume of blood that may lead to thrombosis. Increased expression of glycoproteins, fibrinogen receptors, and P-selectin on the platelet surface promotes platelet aggregation and interaction with white blood cells in order to protect the body from excessive bleeding during the management of acute stress. Mean platelet volume (MPV), which signifies platelet size, is a recognized indicator of platelet activity, while high MPV levels are thought to be strongly associated with cardiovascular disorders [5]. The Conversion And Neuro-inflammation Disorder Observational (CANDO) study, associated the systemic low-grade inflammation with disorders such as conversion disorder (CD), post-traumatic stress disorder (PTSD), depression, etc. [6].

Specific bioactive molecules with strong anti-inflammatory, antioxidant, and antithrombotic activity are an upstanding solution to this matter. There are many anti-inflammatory/oxidant/thrombotic drugs; however, their long-term use has enhanced several unwanted side effects in the past, thus current research has focused on natural bioactives, to avoid the existing disadvantages of synthetic conventional pharmaceutical products. For instance, polyphenols, which are also detected in shrimp, are highly anti-inflammatory constituents because of their regulative role in cytokine production and in the expression of inflammatory genes in the DNA [7]. Similarly, carotenoids, particularly astaxanthin, is abundant in shrimp and it is recognized as a strong antioxidant. Antioxidants are defense mechanisms that halt the formation of free radicals. The imbalance between antioxidants and free radicals leads to oxidative stress that consequently induces inflammation, which in turn may lead both to cell death and the formation of numerous pestering chronic diseases [8].

Lipids consist of an important group of biomolecules essential for our wellbeing, as they can produce large amounts of energy in the form of adenosine triphosphate (ATP) through catabolic pathways, like b-oxidation of fatty acids (FAs), while they may serve as precursors for the synthesis of lipophilic vitamins, such as retinol (Vitamin A) and tocopherols (Vitamin E) [9]. Lipids in general, play a key role in energy storage, signaling, enzymic activity, structure of membranes, etc. During the last decade, marine bioactives have garnered significant interest due to their health-promoting impact coupled with their ample availability. Many studies have been conducted on fish lipids and their effect on several diseases such as cardiovascular diseases, skin issues, and neurological diseases. More specifically, fish n-3 FAs have been proven to improve vascular health, as well as to decrease inflammation, blood pressure, and oxidative stress [10,11,12,13]. Therefore, they have become an integral part of a healthy and nutritional diet, with experts suggesting that people should consume a specific quantity of seafood weekly [14].

Nevertheless, there has been insufficient research on shrimp regarding their bioactive molecules, lipid composition, and overall benefits so far. Aquaculture, the process of breeding, raising, and harvesting fish, shellfish, and aquatic plants, represents a significant advantage of shrimp, due to their cost-effectiveness which enables their mass production. Unfortunately, as of today, during industrial shrimp processing, more than 50% of their components are disposed [15]. Recent trial outcomes indicated the bio-functionality of these by-products and waste can be fully utilized in pharmaceutical, nutraceutical, and cosmetic products, and thus implied that such waste-considered products may promote a more sustainable environment through a circular economy. Nowadays, tons of shrimp waste still ends up being disposed of, triggering environmental problems if it is not carefully handled [16].

Shrimp are naturally rich in FAs, phospholipids, monounsaturated fatty acids (MUFAs), polyunsaturated fatty acids (PUFAs), saturated fatty acids (SFAs) etc. Particularly, important very-long-chain PUFAs (VLC-PUFAs) found in shrimp are docosahexaenoic acid (DHA, 22:6 n-3) and eicosapentaenoic acid (EPA, 20:5 n-3), which are included in n-3 PUFAs. Shrimp’s consumption can nourish the human body with the recommended levels of n-3 FAs, which are associated with cardiovascular improvement. However, both n-3 and n-6 FAs can be synthesized inside the body by the conversion of α-linolenic acid (ALA) and linoleic acid (LA), respectively. Notably, it has been discovered that EPA and DHA composition is increased when a lower quantity of LA is consumed [17]. Such constituents are very important for our body, mainly because we are unable to produce them whatsoever without any previous seafood consumption, and subsequently, due to their high anti-inflammatory and anticoagulant activity, their structural role in all membranes and their function as sources of various metabolites that are linked to the treatment of many tormenting diseases [18].

Apart from FAs, shrimp also contain several other beneficial lipophilic substances, believed to possess antioxidant and anti-inflammatory protective properties towards the cardiovascular system. Many crustaceans, including shrimp, are enriched with antioxidant and anti-inflammatory biomolecules such as carotenoids and phenolics. The most important antioxidant commonly found in relatively high concentrations is astaxanthin, which can be either free or esterified with fatty acids [19]. Astaxanthin (3,3′-dihydroxy-β, β-carotene-4,4′-dione) is a xanthophyll carotenoid with remarkably high antioxidant activity, more than any other carotenoid and even some vitamins.

Shrimp extracts have shown positive effects on other diseases as well, including diabetes, Alzheimer’s disease, obesity, cancer, neurological diseases, etc. Studies have proved that shrimp lipids may exhibit protective action by using restraining compounds so as to stop cancer cell division [20]. Moreover, in vivo studies have demonstrated that n-3 fatty acids (DHA) protects against type II diabetes [21]. Shrimp apparently offer a plethora of benefits that are yet to be fully explored, analyzed, and experimented upon.

The aim of this review is to summarize the lipid composition of many species of shrimp from different areas, and how these lipids can be useful to human health. Emphasis will be given to lipids deriving from shrimp extracts and their effect on cardiovascular diseases, while other pathological ailments and how they can be prevented or cured by lipid-derived natural shrimp products will be further discussed.

## 2. Lipid Composition of Shrimp

Lipids are a class of biomolecules, which can be detected in many cellular processes. Some important functions of these biomolecules entail energy storage, the formation of membrane lipid layers, acting as autocrine/paracrine hormones, etc. [22]. Lipids are commonly divided into two different categories based on their polarity: neutral and polar lipids. Polar lipids consist of phospholipids and glycolipids, which can be observed mostly in cell membranes, because of the ability of such amphiphilic molecules to form bilayers. On the contrary, neutral lipids contain mono/di/triacylglycerols (MAGs, DAGs, and TAGs respectively), waxes, free fatty acids (FFAs), and many more, which are characterized by hydrophobic properties owing to their long hydrocarbon chains and the absence of polar groups. At this point, it is important to mention several significant groups of lipid molecules, such as sterols, particularly cholesterol as well as lipophilic substances, including tocopherols and carotenoids, which are used as pigments in many compounds, like astaxanthin and β-carotenoids.

### 2.1. Total Lipids

Studies on shrimp lipids have confirmed that these species contain a small amount of total lipids (TLs) in comparison to other nutrients like proteins, carbohydrates, etc. More precisely, a higher amount of total crude lipids extracted from shrimp meat, muscle, and oil was obtained from the shrimp *Pleoticus muelleri*, which grows in the North Sea (4.61% of the total extract) [23]. On the other hand, the lowest value of TLs was observed in the meat extract from *Exopalaemon carinicauda* (only 0.43%) [24]. Moreover, in the extracts of shrimp by-products/waste, the highest amount of TLs came from the head and carapace of the *Litopenaeus vannamei* species (7.77%), while the lowest value was found in the head of *Penaeus Monodon* (0.48%) [23,25].

### 2.2. Fatty Acids of Shrimp

FAs refer to molecules with a large aliphatic hydrocarbon chain and a carboxylic acid group. FAs contribute to the structure of cells by being included in cell membranes and serving as a significant energy source, stored in the form of triacylglycerols. Overall, FAs influence cardiovascular diseases through several pathways like cardiac metabolism, lipotoxicity, electromechanical properties of cardiomyocytes, and inflammation [26]. Fatty acids can be divided into saturated (SFAs), monosaturated (MUFAs), and polyunsaturated FAs (PUFA). It is important to mention that MUFAs and PUFAs belong to a broader category called unsaturated fatty acids (UFAs). The content of FAs derived from shrimp extracts of meat and by-products/waste can be seen in Table 1; it appears that FA content varies from one shrimp species to another, but there were significant composition fluctuations even among the same shrimp species. Most researches on shrimp lipid composition emphasize FAs because shrimp are known for having considerable amounts of n-3 PUFAs, in addition to their ability to produce long-chain FAs (LC-FA, 12 to 20 carbon atoms), which cannot be biosynthesized in human cells [27]. Thus, LC-FAs can only be obtained through the consumption of shrimp and other marine consumables. Significant amounts of PUFAs, such DHA and EPA, were noticed. Accordingly, in SFAs, the predominant value was perceived from palmitic acid (16:0, PA), while in MUFAs, the oleic acid (18:1 n-9, OA) was the prevalent fatty acid.

#### 2.2.1. Saturated Fatty Acids in Shrimp

SFAs consist of a long hydrophobic carbon chain that only has single bonds. Saturated FAs also include branched iso- and anteiso-acids, and acids containing a cyclopropane or other fragment without ethylene or acetylene bonds in its structure. As shown in Table 1, the total SFA content of twenty-two shrimp species and shrimp oil meat extracts, ranged from 19.70 to 60.68% of total FAs. The corresponding range obtained from shrimp and shrimp oil by-products was found to be broader (18.09–73.16%). The width of the amounts of SFAs could be explained due to several research results that declared this FAs’ category as prevalent. On the contrary, other studies showed a lower amount of SFAs, and therefore this class was considered secondary after PUFAs. Specifically, the most traced fatty acid in SFAs was PA (C16:0), consisting of 11.28–50.15% and 10.17–32.76% of total FAs in shrimp meat and by-products, respectively. PA reportedly induces inflammatory responses by activating different signal pathways and causes endoplasmic reticulum (ER) stress in high concentrations in macrophages [28]. Other SFAs that did not exhibit significant values (myristic acid (14:0), stearic acid (18:0)), were not included in Table 1.

#### 2.2.2. Unsaturated Fatty Acids in Shrimp

In general, UFAs are large hydrophobic carbon chains composed of one or more double bonds. MUFAs and PUFAs belong in this subclass, and therefore contribute significantly to the total proportion of FAs. Generally, UFAs can break down quicker than SFAs due to the presence of double bonds, which limits their movement. Thus, it is easier for the human body to digest UFAs and receive the health benefits they carry. Additionally, studies have shown that UFAs are closely linked with the improvement of HDL levels. The shrimp meat extract was enriched with a wide range of UFAs from 39.33% of total FAs in *Litopenaeus Vannamei*, to 79.10% in *Pandalus Borealis*. The shrimp by-products had similar UFA amounts, accounting for 36.38–81.90% in shrimp heads of *Penaeus Monodon* and in shrimp oil from by-products of *Pandalus Borealis*, respectively.

#### 2.2.3. Monounsaturated Fatty Acids in Shrimp

MUFAs, are FAs that contain only one double bond in the long hydrocarbon chain. MUFAs are often recommended as a key component of a nutritious Mediterranean diet. A substantial percentage of total FAs is related to MUFAs and ranged from 18.50 to 56.44% of total FAs found in *Litopenaeus vannamei* meat extract. On the other hand, in shrimp by-products and waste, MUFAs exhibited a range from 6.05 up to 40.67% of the total FAs. MUFAs’ class includes important biomolecules such as OA, which accounted for 2.40 to 28.34% of total FAs in shrimp meat and oil. Correspondingly, in by-products and waste, the concentration of OA was traced to about 1.21–22.36% of total FAs.

#### 2.2.4. Polyunsaturated Fatty Acids in Shrimp

The most abundant class of FAs in shrimp was revealed to be the PUFAs, which are composed of more than one double bond in the long hydrocarbon chain. PUFAs can be further subcategorized into n-3 PUFAs and n-6 PUFAs, the most prominent PUFAs for their beneficial action. n-3 PUFAs have one of their double bonds located on the third carbon atom from the methyl end. Importantly, n-3 PUFAs have a positive impact on various cardiometabolic risk factors, with research based on the consumption of fish in the diet proving their cardioprotective benefits. Consuming fish regularly was consistently linked to reduced risks of heart failure, sudden cardiac death, stroke, and myocardial infarction [29]. n-6 PUFAs, on the other hand, have one of their double bonds located on the sixth carbon atom from the methyl end. Extracts of 22 shrimps and shrimp oil from flesh, consisted of 14.55–49.50% PUFAs of total FAs and 16.26–44.10% in by-products and waste of them.

According to Table 1, n-3 PUFAs in general reported higher values than n-6 PUFAs. Shrimp meat contained approximately 12.38–38.63% n-3 PUFAs out of the total FAs and 5.45–37.13% by-products of shrimp, respectively. In addition, studies claim that the range of n-6 PUFAs corresponds to 0.6–25.80% in the meat part of the 22 shrimp species, while a slightly smaller number of total FAs (3.88–30.90%) was found in the by-products/waste. The n-3/n-6 ratio is a representative index to identify a consumable as a good or insufficient source of polyunsaturated FAs. Numerous reports have asserted that balancing the n3/n6 ratio results in the proper functioning of the human body and, more specifically, the enhancement of the proper operation of numerous cellular processes [30,31]. In detail, most shrimp meat extracts and oil depict a range from 0.012 to 8.51. In a study about *Litopenaeus vannamei* meat extracts, it was reported that the n-3/n-6 ratio was 8.51, a case that explains the wide range mentioned above. This index value indicates that n-3 PUFAs were found at higher rates than n-6 PUFAs, due to possible effects that were able to alter the fatty acid content of shrimp, which will be further analyzed in due course. Shrimp by-products and waste showed much smaller values (0.23–4.13) instead. In general, the n-3/n-6 index revealed that the majority of shrimp contain a balanced amount of n-3 and n-6 PUFAs, verifying their reported nutritional value.

Regarding the class of n-3 PUFAs, shrimp meat extract contained DHA as the representative fatty acid at concentrations ranging from 3.06 to 29.66%. However, in shrimp by-products/waste, it was observed that DHA was not the essential fatty acid of n-3 PUFAs (4.57–17.39%), but instead, EPA was traced at higher amounts (3.08–21.78%). In the shrimp flesh, EPA appeared to be the second most abundant n-3 PUFA, with relatively significant values in almost all 22 shrimp species (5.73–17.90%). EPA and DHA, allegedly, are two very important FAs capable of impacting various cardiovascular functions like inflammation, anticoagulation, coronary incidents, etc. [18]. Even though ALA, a major n-3 PUFA, was not observed at a high percentage in most shrimp, it is an important FA for humans, due to our lack of the ability to produce it. In shrimp meat extracts and by-products, the percentage of ALA was reported as 0.0–9.75% and 0.01–1.50%, respectively.

Moving on to n-6 PUFAs, a remarkable FA that belongs in this category is LA. Research has shown that LA, is correlated with lower mortality rates from causes including cardiovascular diseases, cancer, and diabetes [32,33]. Hence, the LA extract showed a wide range in both shrimp meat (0.60–20.10%) and in shrimp by-products (0.01–24.60%). *Litopenaeus vannamei* was linked to the highest amount from meat extract (20.10%) and shrimp waste (24.60%). Additionally, it was found that *Pandalus Borealis* had the lowest value of LA in shrimp meat (0.60%), similarly to the river prawn *Macrobrachium rosenbergii* in shrimp by-products (0.01%). Arachidonic acid (20:4 n-6, ARA), the predominant n-6 FA, is converted to prostaglandins, leukotrienes, and other lipoxygenase or cyclooxygenase products, which regulate inflammation, prothrombosis, and atherogenesis [34]. The concentration was determined in shrimp meat and by-products to range from 0.40 to 17.34% and 0.04 to 8.72%, respectively, while the highest percentage (17.34%) was detected in *M. monoceros* meat extract.

**Table 1:** Composition of FAs (%) of total FAs in meat/flesh and by-products/waste of different shrimp species.

	Shrimp Families	Species (% of Total FAs)	16:0	18:1 (n-9)	18:3 (n-3)	18:2 (n-6)	20:4 (n-6)	20:5 (n-3)	22:6 (n-3)	SFAs	MUFAs	PUFAs	n-3PUFAs	n-6PUFAs	n3/n6	TLs g/100 g	References
Meat/flesh	*Penaeidae*	*L. vannamei*	12.57–50.15	8.22–23.8	0.61–6.28	7.76–20.1	1.53–4.92	5.73–17.47	3.06–16.95	21.16–60.68	18.50–56.44	14.55–48.40	12.38–22.60	1.53–25.80	0.88–8.51	0.71–4.50	[23,24,35,36,37,38,39]
		*M. kerathurus*	13.25–17.99	9.59–15.80	0.35–0.70	1.38–6.25	1.0–11.04	6.40–17.28	13.05–17.90	28.80–35.0	23.54–25.50	39.50–45.35	25.00–34.69	3.60–13.58	2.34–6.94	1.01–1.50	[40,41,42,43]
		*P. semisulcatus*	16.82–18.26	17.10–17.70	0.07–0.13	0.91–1.83	7.59–8.51	11.71–12.65	10.78–12.30	33.28–38.14	29.05–32.65	30.89–35.99	23.51–23.59	9.81–9.97	2.39	0.51–1.57	[43]
		*P. monodon*	16.8–20.37	14.6–19.68	0.00–9.75	3.79 -13.54	3.35–8.88	10.1–14.62	10.5–10.81	34.0–35.84	21.44–23.50	42.50–42.72	23.36–24.20	17.89–18.30	1.29–1.35	1.17–4.60	[23,36,37]
		*P. japonicus*	16.40–18.36	18.62–20.24	0.35–0.55	7.89–9.23	4.02–5.40	10.73–12.59	11.70–13.44	29.97–33.89	28.3–31.34	34.90–41.60	22.97–26.39	12.13–15.01	1.82	0.87–2.15	[43]
		*F. aztecus*	11.28–22.77	13.55–13.76	0.70–0.81	2.49–5.38	6.24–7.66	9.05–14.36	7.62–29.66	36.42–51.54	23.65–32.61	25.89–34.57	15.15–21.21	10.74–13.36	0.60–1.75	0.51–0.94	[37,44]
		*F. chinensis*	16.60–17.40	10.20–11.20	1.10–1.30	10.60–12.20	2.10–2.50	14.10–16.30	14.10–15.70	26.30–29.70	18.80–20.0	45.50–49.50	30.00–32.80	15.30–16.70	1.90–2.1	1.10–1.26	[24,25]
		*P. longirostris*	19.30–21.16	5.50–18.48	0.12–0.60	1.11–2.20	4.50–5.44	8.70–15.0	13.60–19.50	33.20–35.73	22.90–30.32	33.95–44.00	22.90–36.00	6.93–8.00	3.18–4.60	1.33–1.81	[41,43,45]
		*M. monoceros*	13.38–15.16	9.57–12.39	0.12–0.22	1.50–2.22	14.60–17.34	11.91–12.67	10.85–11.85	29.27–37.23	20.35–27.59	39.98–45.56	22.84–24.76	17.86–20.04	1.26	1.58–2.74	[43]
	*Pandalidae*	*P. borealis*	12.50–14.90	12.30–12.90	0.60	0.60–0.80	0.40	10.40–12.50	7.10–7.70	19.70–21.40	46.0–49.90	25.30–29.20	21.70–25.20	0.60–1.70	-	-	[46]
		*P. martia*	19.03–22.00	2.40–23.68	0.25–2.40	2.9–3.15	3.02–3.40	6.00–9.72	16.06–20.20	29.93–38.90	21.90–37.87	32.20–39.20	25.55–26.51	6.08–6.26	0.01	-	[41,43]
		*P. edwardsii*	18.76–20.62	23.45–24.41	0.17–0.23	2.08–2.58	3.43–4.27	9.16–10.38	14.59–16.14	27.37–31.79	37.78–40.00	29.42–33.64	25.20–25.50	6.02–6.34	4.11	-	[43]
	*Aristeidae*	*A. foliacea*	19.26–19.76	21.99–23.43	0.49–0.51	1.01–1.17	3.23–3.25	10.03–10.71	18.27–18.33	30.34–32.04	33.40–36.44	33.38–34.38	29.03–29.79	4.41–4.55	6.58	1.45–2.97	[43,47]
		*A. antennatus*	20.35–21.23	21.96–22.54	0.43–0.71	0.77–1.09	3.34–4.06	8.42–10.36	13.56–16.40	31.19–34.27	35.29–39.89	26.84–32.52	22.51–26.61	4.53–6.11	4.808	2.24–2.48	[43]
	*Crangonidae*	*C. crangon*	27.38	8.00	1.85	7.53	3.85	5.95	14.75	36.49	26.30	37.21	23.35	13.56	0.52–0.62	1.35–1.45	[48]
	*Solenoceridae*	*P. muelleri*	18.33–18.67	18.55–18.85	-	1.28 –1.48	3.43–3.49	15.56–16.04	19.92–20.48	26.17–27.23	28.25–28.80	44.51–45.49	37.77–38.63	6.61–6.93	4.03–5.65	3.65–4.61	[23]
	*Palaemonidae*	*M. rosenbergii*	17.40–19.40	12.20–14.00	1.30–1.50	15.30–17.50	4.50–5.50	10.70–12.50	4.70–5.10	27.50–30.30	24.60–26.40	39.60–43.20	17.70–18.90	21.80–24.40	0.70–0.90	1.78–1.94	[24]
		*M. nipponense*	25.10–27.70	16.40–18.02	1.90–2.10	8.20–9.20	3.60–4.00	7.50–8.70	6.40–7.20	36.30–40.50	24.60–26.40	31.20–34.40	16.40–18.00	15.00–16.40	1.10	1.23–1.43	[24]
		*E. carinicauda*	15.30–16.50	11.70–12.50	2.40–3.00	3.30–4.10	2.90–3.10	16.50–17.90	10.80–12.00	24.30–26.70	27.50–31.10	37.80–40.40	30.60–32.40	7.10–7.90	4.00–4.40	0.43–0.49	[24]
		*E. annandalei*	15.20–17.40	13.00–14.00	1.00–1.20	1.20–1.40	6.20–7.00	12.90–14.70	14.20–16.20	28.20–31.20	24.10–26.70	37.20–41.80	28.80–31.40	9.00–9.80	2.90–3.50	1.66–1.90	[24]
	*Odontodactylidae*	*S. mantis*	14.26–15.50	7.78–10.44	0.37–0.57	0.89–1.81	6.02–7.72	12.68–14.86	12.18–14.60	27.86–28.76	25.23–28.35	36.82–43.56	27.92–32.24	6.89–9.79	3.30–3.90	0.77–1.33	[40]
		*O. oratoria*	13.70–15.10	11.50–13.50	1.90–2.50	1.60–1.80	4.60–5.40	12.00–14.40	15.10–16.90	24.60–26.60	24.60–27.20	40.00–42.60	30.40–34.00	8.50–9.70	3.30–3.70	1.33–1.51	[24]
By–products/waste	*Penaeidae*	*L. vannamei*	10.17–32.76	7.77–21.40	0.02–0.76	8.55 –24.60	0.33–5.04	4.69–9.44	4.57–16.25	27.40–62.65	13.72–28.50	23.60–44.10	13.20–26.79	15.98–30.90	0.43–0.73	3.78–7.77	[23,25,36,49,50]
		*M. kerathurus*	17.07–17.33	9.26–9.56	0.38–0.42	1.05–1.09	8.58–8.72	10.58–10.82	10.27–10.59	42.23–42.57	22.23–22.57	34.88–35.32	22.56–22.82	12.25–12.57	1.80–1.86	2.35–2.45	[42,51]
		*P. monodon*	16.90–20.60	3.29–14.90	0.04	3.46–11.04	0.13–7.20	7.47–9.85	6.15–6.44	38.70–63.63	6.05–26.70	30.33–34.60	15.79–17.21	17.92–18.28	0.91–1.20	0.48–5.95	[23,25]
		*P. japonicus*	30.02–30.06	5.71	0.03	0.82	0.04	7.25	4.33	73.16	10.58	16.26	-	-	2.70	2.14–2.20	[25]
		*F. chinensis*	31.85–31.87	12.65–12.67	0.01	10.83	0.69	3.08	4.90	62.35	15.83	21.8	-	-	0.595	3.85–3.91	[25]
		*P. longirostris*	12.13–14.73	1.21–1.39	-	0.78–0.92	-	16.50–21.78	-	33.87–35.49	30.90–32.44	21.98–22.98	5.45–5.83	21.11–22.93	0.23–0.29	3.55–4.39	[52]
	*Pandalidae*	*P. borealis*	15.40–16.06	20.30–22.36	0.21–0.39	1.84–2.08	1.55–1.83	21.09–21.21	13.76–14.02	18.09–18.59	40.49–40.67	-	37.05–37.13	3.88–4.10	-	-	[19]
	*Crangonidae*	*C. crangon*	19.90	14.10	1.50	1.30	2.20	11.90	5.10	29.20	42.40	27.80	22.10	5.40	4.09	7.40	[53]
	*Solenoceridae*	*P. muelleri*	16.04–16.36	17.60–18.0	-	1.44–1.54	4.15–4.31	14.04–14.16	17.01–17.39	25.09–25.31	31.66–32.34	42.36–43.24	34.04–34.56	8.26–8.74	3.93–4.13	4.07–5.15	[23]
	*Palaemonidae*	*M. rosenbergii*	18.18–18.22	3.17	0.26	0.01	1.99	10.29	5.47	61.54	9.63	28.70	-	-	1.33	6.83–6.93	[25]

### 2.3. Neutral Lipid Content in Shrimp

The main neutral lipids that can be spotted in shrimp are TAGs, sterols, and, in smaller proportion, waxes, etc. Such lipids play a major role in membranes, the trafficking of lipoproteins, steroidogenesis, etc. [54]. Neutral lipid composition in the prawn *Melicertus kerathurus* exhibited values of 22.5–22.7% (of TLs) in its meat extract, while in the by-products the difference was significant (51.1–51.5%). *Litopenaeus Vannamei* meat and by-products extracts contained a percentage of 1–1.28% and 2.23%, respectively, of its TLs in TAGS, while *Melicertus Kerathurus* had an even bigger percentage, varying from 7.9–8.1% and 22.93–22.96% of TAGS, respectively, as shown in Table 2. The TAG percentage, however, is highly correlated with shrimp diet, a situation that clarifies the big difference, as they serve a great role in energy saving and resourcing [42]. Monoacylglycerols and diacylglycerols appeared to have a much lower percentage of 0.34 and 0.43–2.2% of TLs in *Litopenaeus Vannamei* meat, respectively. *Penaeus Monodon* seems to be in the middle ground and had a percentage of 2.27–2.69% TAGs and lower amounts of MAGs and DAGs. However, the amount of this neutral lipid subcategory depends on the diet of the shrimp and, more specifically, its growing conditions. At this point, it must be noted that waxes are long-chain FAs connected to an alcohol through an ester of oxygen, while they are not soluble in water and can even be found in solid form at environmental temperatures. Waxes serve as energy storage units for marine animals. The percentage of *Melicertus kerathurus* waxes ranges from 0 to 0.1% of TLs, while *Pandalus borealis* by-products showed higher amounts (0.58–0.76%) of TLs. Nonetheless, waxes have not been thoroughly studied in other species.

### 2.4. Polar Lipid Content in Shrimp

Polar lipids (PLs) are structural elements of all cell membranes. Glycerophospholipids, otherwise phospholipids, are a class of PLs that possess a phosphate group in the polar head and two hydrophobic tails comprised of FAs and alcohols. These lipids have a very important role in metabolism, cell signaling, lipid layers, etc. [56]. Subsequently, phospholipids are divided thoroughly into Phosphatidylcholine (PtdCho), Phosphoethanolamine (PtdEtn), Phosphatidylinositol (PtdIns), and Phosphatidylserine (PS). It is worth mentioning that there was limited research conducted on the PL composition in shrimp with emphasis given in *Litopenaeus vannamei*. The abundance of PLs was found to be 77.02–77.56% of the TL content in *Litopenaeus vannamei* meat extract. Moreover, in the by-products of the same shrimp, the amount of PLs was similar (67.09–68.71%). Phospholipid content was analyzed only in shrimp by-products, varying from 47.10 to 68.80% of PLs. Additionally, glycerophospholipids were measured in *Litopenaeus vannamei* and *Penaeus Monodon* meat extracts (74.40–77.29% of TLs), and in *Litopenaeus vannamei* by-products/waste (67.90%). In previous studies that calculated the subclasses of phospholipids, it appeared that PtdCho was the prevalent phospholipid (meat: 52.80%, by-products/waste: 46.80–47.60% of total PLs), while PtdEtn came in second (meat: 24.70%, by-products/waste: 24.5–24.9%). Moreover, PtdIns value was found only in one research about meat extracts of *Litopenaeus vannamei* (1.10%), while the PS value was not discussed or further analyzed. All obtained results are summarized in Table 3.

### 2.5. Marine-Derived Bioactives

Carotenoids are a common group of pigments observed in marine environments and land organisms. Marine autotrophic organisms such as bacteria, algae, fungi etc. can biosynthesize these biomolecules, while heterotrophic organisms can retrieve carotenoids from their food. Carotenoids may also be modified through metabolic reactions and can be divided into carotenes, hydrogen and carbon substances, and xanthophylls, which include oxygen as well. These compounds are known for their antioxidant properties, and more specifically for quenching singlet oxygen, absorbing light, and protecting photosynthesis [57]. Thus, various health benefits from these bioactives have been reported, such as anticancer, anti-obesity, antidiabetic, anti-inflammatory, and cardioprotective activities [58]. Astaxanthin, as previously mentioned, is the most common xanthophyll biomolecule in crustaceans, especially in shrimp, as it occurs through the oxidation of β-carotene. This xanthophyll type has high antioxidant activity, more than any other carotenoid, and it is responsible for the orange color of shrimp [59]. Notably, it can be found even in the waste of the shrimp, such as heads, tails, etc., and it can be extracted by many solvents like vegetable oils, methyl esters, or organic solvents. Hence, studies have focused their research regarding astaxanthin on shrimp by-products. In shrimp by-products, astaxanthin accounted for 1.8–19.20 μg/g of the total shrimp part studied. In parallel, *Litopenaeus vannamei* showed a much lower content of astaxanthin (0.69–0.77 μg/g) in its meat extract. β-carotene, pro-vitamin A, belongs to the family of carotenes and is the second most common carotenoid biomolecule found in shrimp species. Nevertheless, its concentration is significantly lower compared to astaxanthin, which is why many researchers do not focus on exploring its composition, a hypothesis further supported by studies in which the meat extract of *Litopenaeus vannamei* contained 0.05–0.013 μg/g of β-carotene in the total shrimp meat [59].

**Figure 1:**
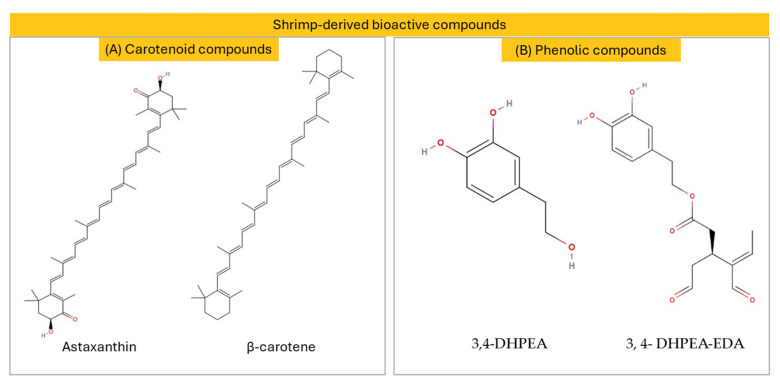
The structures of the most abundant carotenoids (**A**) and phenolic compounds (**B**) present in shrimp.

Phenols consist of a hydroxyl group which is bonded with an aromatic hydrocarbon group, able to inhibit enzymes that are associated with human diseases. Marine-derived phenols are far less studied than terrestrial phenols, due to the complexity and variability of their structures, which demand advanced analytical methods [60]. Phenolics and polyphenolic compounds serve as antioxidants, protecting the tissues of the human body from being damaged by oxidative stress. Furthermore, they can be applied in various therapeutic purposes because of their impact on inflammation [61]. The total phenolic content was observed to be lower in *Pandalus borealis* flesh extract (0.19 mg of gallic acid equivalents (GAE)/g) and by-products (0.18–0.28 mg GAE/g), relative to *Palaemon serratus* and *Palaemon varians* by-products (4.4–11.1 mg GAE/g) [62,63]. Nevertheless, a study focusing on quantifying phenolic compounds in *Parapenaeus longirostris* found that 3,4-dihydroxyphenylethanol (3,4-DHPEA), was the most abundant polyphenol in raw shrimp (249.74–250.62 mg/kg) that were treated with a tap water solution that contained phenols (2 g/L). Another notable polyphenol was 3, 4-(dihydroxyphenyl)ethanol (3, 4- DHPEA-EDA), also known as oleacein, which was found at a percentage of 223.36–231.36 mg/kg, which, along with 3,4-DHPEA, is a beneficial phenolic compound for the cardiovascular system and a part of the Mediterranean diet [64]. These bioactives, with their endothelium-dependent relaxing effects, have demonstrated a beneficial vasorelaxant effect in in vivo studies [65,66]. In Figure 1, the structures of the most abundant carotenoids (Figure 1A) and phenolic compounds (Figure 1B) present in shrimp are represented.

Tocopherols, the major forms of vitamin E, are fat-soluble phenolic compounds that are known for their antioxidant and anti-inflammatory effects. More specifically, vitamin E is a powerful antioxidant that disrupts the formation of reactive oxygen species when fat is oxidized and during the spread of free radical reactions [67]. Tocopherols possess great health benefits, such as the prevention of cancer, heart diseases, and other chronic pathologies. Important functions of tocopherols include the regulation of gene expression, signal transduction, and adjustment of cell functions [68]. An investigation which examined the concentration of tocopherol in shrimp flesh revealed that *Pleoticus muelleri* and *Litopenaeus vannamei* contain an α-tocopherol value of 16.4–16.7 μg/g of fresh-weight meat extract, while *Penaeus Monodon* value was traced at 21.7 μg/g. The concentration of α-tocopherol in the by-products from shrimp species was elevated, reaching 35.3 μg/g of fresh weight [23].

Marine sterols belong to the complex of sterol compounds, which are lipidic compounds that contain a steroid nucleus with a hydroxyl group at the 3rd carbon and a side chain at the 17th carbon. Moreover, these molecules have an essential contribution to human health, via promoting the protection against oxidative stress, apoptosis and also neuroinflammation [69]. Sterols were found to be present in lower quantities in the meat extract of Mediterranean prawn *Melicertus kerathurus* (146.78–173.02 mg/100 g fresh weight), compared to the by-products of the prawn (520.9–539.9 mg/100 g fresh weight (FW)). A similar result was obtained from a study on *Pleoticus muelleri* meat, providing equal levels of steroids (118.13–126.27 mg/100 g). It is noteworthy that sterols can be chemically bonded with other groups to form sterol esters. These bioactives were studied in *Melicertus kerathurus* meat and by-products and were found to be minor substances contained in TLs (0.2–0.4 and 1.9–2.3% of TLs, respectively).

Cholesterol, the principal sterol of shrimp, is a crucial bioactive found in cell membranes and biosynthesized by all animal cells. The predominant role of this substance is to uphold the integrity and flexibility of cell membranes, although it can also act as a precursor for the synthesis of steroid hormones, vitamin D, and other important biomolecules [70]. Cholesterol is distributed in tissues, the brain, fats, oils, etc., and is highly affected by the diet of the shrimp. The cholesterol concentration in *Litopenaeus Vannamei* meat extract was identified as approximately 34.94 mg/100 g FW and 50.47 mg/100 g, whereas in *Penaeus Monodon*, it exhibited values 40.91 mg/100 g and 55.93 mg/100 g, respectively. The cholesterol level in the meat and by-products of the prawn *Melicertus kerathurus* was found to be at a higher concentration (130.21–158.37 and 511.81–531.07 mg/10 g, respectively). All obtained results are included in Table 4.

### 2.6. Effects in Shrimp Lipid Composition Variations

The lipid composition, as shown in all tables, is not the same for all shrimp but is influenced by endogenous and exogenous factors. The most important factors will be further discussed in detail in the following sections.

#### 2.6.1. Effects of Sex in Shrimp Lipid Composition

A plethora of studies in the past showed a relevance between the shrimp’s sex and its lipid composition. Specifically, a study conducted on the species *Harpiosquilla harpax* and *Miyakea nepa* showed that females of the former consisted of more SFAs with a percentage of 48.37–49.70%, while males possessed a lower percentage of 44.65–46.76%. In parallel, for the species *Miyakea nepa*, female shrimp were comprised of 47.09–48.72% SFAs and males followed with 45.61–47.81%. A similar pattern is noticed considering MUFAs, with female *Harpiosquilla harpax* shrimp having a bigger percentage (18.38–20.82%) in contrast to males (15.28–19.23%) and *Miyakea nepa* shrimp as well (females: 18.75–20.25%, males: 17.88–20.14%). However, males seem to have a bigger portion of PUFAs than females in both species, specifically regarding their DHA and EPA concentrations. *Harpiosquilla harpax* female shrimp consisted of 7.59–9.76% EPA and 10.50–12.03% DHA, while at the same time male shrimp showed bigger values of 9.56–12.50% EPA and 11.84–13.85% DHA. *Miyakea nepa’s* males had higher amounts of DHA than females (males: 10.28–12.13%, females: 8.80–10.11%), but lower amounts of EPA (males: 8.53–10.65%, females: 9.09–12.18%).

Such differences in the lipid composition of shrimp, can be further explained by the differences following each gender. The biosynthesis, metabolism, and hormones of each shrimp are major variables considering the lipid composition. Hormones can also affect the metabolism of FAs, as shown by their lipid profile. Specific interest is also warranted for PA, due to its connection with the metabolic energy during egg formation for female shrimp, which can explain why both female species have higher amounts of C16:0 (approximately 26 to 29%) than males (approximately 22 to 26%) [74]. Another study worth mentioning on the *Plesionika semilaevis* species exhibited that female shrimp have partially higher lipid content than males. Non-berried female shrimp have a total amount of 36.18–36.54% of SFAs and 22.86–25.52% of MUFAs, while males exhibit lower ratios consisting of 30.13–31.47% of SFAs and 20.03–22.43% of MUFAs. However, male shrimp PUFAs remain higher (44.30–49.10%) than the corresponding amount in females (37.1–38.4%). All observed differences are attributed to the necessary physical needs of females to be able to reproduce, as body conformation and constitution certainly change [75]. Additionally, PtdCho was observed to be in higher amounts in a morphotype of male *Macrobrachium rosenbergii*, which was in a transitional stage, and the lowest concentration was again obtained from males of this shrimp species, in a different morphotype this time [76]. All things considered, it is important to gather more data in order to fully evaluate the effects of sex on the lipid composition of shrimp.

#### 2.6.2. Effect of Season on Shrimp Lipid Composition

Studies have proven that, during cold weather, PUFAs increase in the lipid composition of shrimp compared to warmer periods. This usually happens because the membranes of the body need different fluidity and structure in order to survive due to cold waters, and at the same time enzymes tend to be more active so as to desaturate in these temperatures. This is proved by the analysis of the *Harpiosquilla harpax* species mentioned before, where in February n-3PUFAs were obtained at a percentage of 28.26%, while in June this value dropped to 23.79% [74]. This hypothesis was also confirmed through another combined analysis of the species *P. serratus* and *P. varians* that were caught in Matosinhos, Portugal, which demonstrated that in the autumn of 2017 when the weather was colder, PUFAs had a percentage of 0.294–0.402%, while in the spring of 2018 when the weather was hotter, PUFAs were found at 0.26–0.268% of the shrimp’s total FA amounts [77]. Thus, in order for a living organism to protect itself during seasonal changes in temperatures, lipids are in a position to ensure the survival of the organism, via changes occurring in the membrane composition.

#### 2.6.3. Effects of Egg-Bearing in Shrimp’s Lipid Composition

During the process of egg-bearing, the lipid composition of a shrimp alters. A study on Mantis Shrimp showed that non-egg–bearing shrimp had a bigger amount of PUFAs (38–40% of total FAs) and MUFAs (49–59%) than egg-bearing shrimp, which during this period PUFAs were at 34.9–35.1% and MUFAs at 49–51%. Indeed, the observed decrease in PUFAs in shrimp during the reproductive phase is associated with the lipid composition of their eggs and the timing of egg production. Throughout the development of eggs, shrimp mobilize their lipid reserves, particularly phospholipids rich in PUFAs, to support adequate embryonic development. This physiological transfer leads to a reduction in PUFA levels within the muscle tissue of the shrimp. Furthermore, seasonal variations significantly impact the PUFA content in shrimp due to climatic conditions, such as temperature and the seasonal availability of dietary sources rich in PUFAs. However, a difference is noticed in the total SFAs during that change, where non-egg-bearing shrimp had a percentage of 4–4.6% SFAs, while egg-bearing ones had a much higher amount of 10.6–11.4%. A big difference is noticed in the amount of stearic acid as well, before (0.6–1%) and after egg-bearing (7.1–7.9%) [78]. On the contrary, another conducted study displayed the opposite effect, with *Plesionika semilaevis* possessing lower amounts of SFAs in egg-bearing shrimp (33.02%) than that recorded in non-egg-bearing shrimp (36.36%). In this case, PA was the main SFA found in non-egg-bearing shrimp in the pre-monsoon (reproductive) period, with an amount of 24.6%, while egg-bearing shrimp had it in lower amounts (23.30%). This fact is associated with the reproduction needs of the female shrimp, leading to a suggestion that more energy is required during egg formation, or that ARA reproduction is also important for female shrimp, due to the fact that it partakes as a major factor in producing prostaglandin hormones needed for reproduction. Female shrimp showed an amount of 3.3% ARA before and after the egg-bearing [75]. The reproduction factor seems to possess a co-dependent role with the shrimp habitat and the period of analysis, and hence widely affects the lipid composition of female shrimp.

#### 2.6.4. Effects of Diet on Shrimp’s Lipid Composition

Food dietary pattern and consumption plays a major role in the shrimp’s lipid composition. An experiment that was conducted on *Litopenaeus vannamei*, a ubiquitous shrimp in the analysis field, displayed some interesting outcomes. Shrimp were fed with four different diets consisting of different percentages of fish meal and freeze-dried powder of the Antarctic krill *Euphausia superba* (FDPE). The first diet consisted of only fish meal (35%) and was used as a control to evaluate better the upcoming results. While on the first diet, shrimp had normal amounts of lipids in their hepatopancreas, with PUFAs having the biggest percentage of 43.05% of total FAs, followed by SFAs with a percentage of 31.33%, and lastly MUFAs with 25.50%. PA was the main SFA, with an amount of 19.53% of its total FA composition. With diet number two (10% FDPE and 25% fish meal), there was a reduction in SFAs compared to diet number one, leading to a percentage of 29.55%, with PA still being the main component. However, a rise was observed in MUFAs (from 25.50% to 30.61%), with PA again having the biggest percentage at 20.43%. PUFA amounts were lower in comparison to the first diet, which only consisted of fish meat (from 43.05% to 39.73%). As the FDPE percentage increased and the fish meat amount decreased, in the following two diets MUFAs were significantly higher and PUFAs decreased in comparison to diet number one. Moreover, PUFAs in the second diet were much higher than the other two diets. EPA and DHA were found at a higher percentage in the first control diet in contrast to the other diets.

As a conclusion, a more lipid-based diet affects all the lipid levels, due to the fact that krill have an almost similar composition to shrimp, as it specifically raises the MUFAs lipids and lowers the PUFAs in the hepatopancreas of the shrimp. In the muscle of the shrimp, however, a different pattern was recorded. Neither MUFAs and PUFAs nor SFAs were dramatically affected by the dietary pattern. The only significant observation was that their lowest percentage was noticed in the fourth diet, which consisted of 30% FDPE and 5% fish meat (35.47%). During the same diet, EPA seemed to have a slightly bigger percentage than at the beginning, whereas DHA had a lower one. It is obvious, however, that diet affects the lipid composition of shrimp [79]. Another study supported these findings as when *Litopenaeus Vannamei* shrimp were fed with LA and ARA, an increase in the DHA and EPA levels in their muscles and hepatopancreas was observed. Therefore, not only does diet directly affect the lipid composition of shrimp, but also by inducing the right modifications in bred shrimp, we may profit from the beneficial increase in necessary lipids [80].

#### 2.6.5. Effects of Extraction and Cooking Method on Shrimp’s Lipid Composition

Lipid composition on a shrimp intended for consumption or analysis is stable; however, we can rarely retrieve 100% yield. Considering shrimp destined for analysis, the extraction method and solvent for the sample play a major role in the awaited results. A study conducted on shrimp by-products deemed to be wasted showed that different organic solvents retrieved different quantities of lipids. Ethanol was the best used solvent in this case, rather than other solvents such as n-hexane and acetone, due to its high dielectric constant, C^2^/(N × M^2^) (where C: Coulomb, N: Newtons, and M^2^: square meters). Polar solvents, such as ethanol, and many others with a bigger dielectric constant than non-polar solvents are a better option for the extraction of polar components found in the shrimp, including phospholipids. Extraction yield is also much higher when choosing these solvents. FAs are a bit more complicated in terms of retrieving them because, depending on the molecule, different solvents should be used due to different polarities. SFAs, for instance, were better extracted with n-hexane, as their long-saturated carbon chain provided them with a lower polar character. Additionally, MUFAs were better extracted with acetone and ethanol due to their double bond, which provided an imbalance in electrical charges and the corresponding polarity, while PUFAs were retrieved in a higher yield by utilizing ethanol, as they had much bigger polarity than MUFAs due to their multiple double bonds [81].

Regarding shrimp consumption, the goal is to get as much as possible beneficial lipids from them. Preparing and cooking via different methods affects shrimp’s lipid composition. An experiment conducted on *Penaeus notialis* showed that SFAs of fresh shrimp (31.09%) increased when it was sun-dried (32.83%), but decreased when it was smoked (30.27%). MUFAs were at peak concentrations (40.35%) when smoked, while the fresh shrimp had 39.65% MUFAs and a big reduction occurred after the shrimp were sun-dried (35.87%). On the other hand, PUFAs, the most valuable lipids in controlled portions, consisted of 31.46% after being smoked, with 4.40% being recorded in n-6 PUFAs and the rest (24.97%) in n-3 PUFAs. Thus, an acceptable n-3/n-6 ratio was achieved, which implicated that they were not competitive against each other. In addition, PA had the lowest percentage when it was smoked (19.05%). This outcome is linked to many advantages, since PA is known for raising cholesterol levels. Smoked shrimp also seemed to have satisfying quantities of oleic and stearic acid, which are related to improving the cardiovascular system’s health. Last but not least, EPA’s highest percentage was spotted in the sun-dried samples (18.50%), while the values of smoked shrimp were much lower than the initial sample, due to induced oxidation from the heat of smoking [82]. Hence, the cooking of the shrimp plays an important role in the yield of retrieved lipids.

#### 2.6.6. Effect of Environmental Conditions (pH, Temperature) on Shrimp Lipid Composition

The pH of the water seems to also affect the fatty acid composition of shrimp. A study conducted on *P. elegans* proved that different pH levels of water combined with different temperatures drastically affected lipid composition. At a temperature of 18 °C, shrimp had bigger amounts of SFAs and lower amounts of PUFAs, while MUFAs remained the same in a more alkaline pH level of 8.1 compared to waters with pH = 7.8. In higher temperatures (22 °C), a similar pattern was noticed; however, MUFAs displayed a lower percentage. Warmer waters also lead to a decrease in the DHA and EPA percentage, a phenomenon primarily noticed in waters with lower pH values. At 22 °C, for shrimp raised in alkaline water of pH = 8.1, PUFAs were found at a percentage of 33.7%, while in pH = 7.8 they were found correspondingly at 32.3%. As indicated, for lower pH values, shrimp enhanced their membrane fluidity so as to enable an exchange between ions across the membrane bilayers, in order to control their acid–base balance [83]. Thereby, pH levels have a direct effect on the membranes’ reactions, and thus a shrimp’s lipid composition may undergo to structural changes with a view to maintain the pre-existing equilibrium.

#### 2.6.7. Effect of the Geographical Position of the Ocean on the Lipid Composition of Shrimp

The living conditions of shrimp significantly impact their biochemical composition, including their lipid content, as already mentioned above. Climate, shrimp habitat type, and nutrient availability are primary factors affecting shrimp composition. In particular, seawater salinity, combined with the temperature variations that characterize different marine environments, play a critical role in shaping the composition of shrimp and other aquatic organisms. Concerning salinity, different shrimp species in different oceans exhibit energy reorganization processes that facilitate osmoregulation, thus maintaining both intracellular and extracellular osmotic balance in response to the different levels of salinity found in their respective habitats [84]. However, the primary criterion for this analysis is the temperature of the different seas, with observations showing temperatures as low as −1.8 °C in the Arctic Ocean, while maximum temperatures in the Pacific and Atlantic Oceans can reach up to 27 °C, depending on seasonal variations.

Using the equator as a reference, it is imperative to assess differences in lipid composition based on the geographical location of the oceans. Obviously, there will be distinct differences in the biochemical composition of shrimp collected from cold waters such as the Arctic Ocean, for instance, compared to those assembled from warmer waters such as the Pacific or Atlantic Ocean, especially those closer to the equator. Research carried out on Crangon reproduction shrimp from the Baltic Sea—where temperatures range between −3 °C in winter and 18 °C in summer—revealed that the highest percentages of n-3 and n-6 polyunsaturated fatty acids (PUFAs) in the total fatty acid content occur during the summer months, with values reaching 42% and 7%, respectively. In contrast, winter analyses showed the lowest percentages, with values of 9.1% for n-3 PUFAs and 3.1% for n-6 PUFAs. It is important to note that there is an inverse relationship in terms of the percentage of MUFAs within the total fatty acid content. The highest percentage of MUFAs is recorded during the winter months (32.5%), while the lowest percentage is observed in spring (22.8%) [85].

The variability of lipid composition affected by temperature is evident and correlates with the geographical position of the ocean. Focusing on the Indian Ocean, particularly along the west coast of Peninsular Malaysia, where water temperatures range from 28.3 to 29.9 °C and represent the highest temperatures found in this ocean, it is important to mention an analysis performed on two shrimp species, *Harpiosquilla harpax* and *Miyakea nepa*. In these species, maximum PUFA values were recorded in February, ranging from 35% to 36%, while maximum MUFA values were observed in June, ranging from 12% to 13% of total fatty acids. The fact that the maximum PUFA values were observed in February and not in June can be explained by the small difference in water temperature between these two months [74]. However, a remarkable phenomenon was observed in cold water environments, such as the Baltic Sea, where the proportion of PUFAs relative to total fatty acids significantly increased compared to warmer waters. Excluding the different shrimp species and their specific characteristics, this observation can be fundamentally understood with latitude. In colder ecosystems, organisms require higher PUFA concentrations to maintain cell membrane fluidity at lower temperatures. The structural characteristics of PUFAs, characterized by multiple double bonds, prevent tight packing of fatty acid chains, thus ensure membrane flexibility. In comparison, in warmer environments, where elevated temperatures naturally enhance membrane fluidity, organisms tend to require fewer PUFAs and a higher proportion of SFAs and MUFAs to maintain the structural integrity of their membranes [77]. To conclude, it can be argued that as the proximity to the equator increases for the shrimp selected and assigned for lipid composition analysis, there will be a corresponding decrease in PUFA content. This phenomenon is mainly attributed to the prevailing climatic conditions and the need for survival of the species. In contrast, shrimp living in colder climates have reportedly exhibited higher PUFA levels.

## 3. Cardioprotective Properties of Shrimp Lipid Bioactives

**Figure 2:**
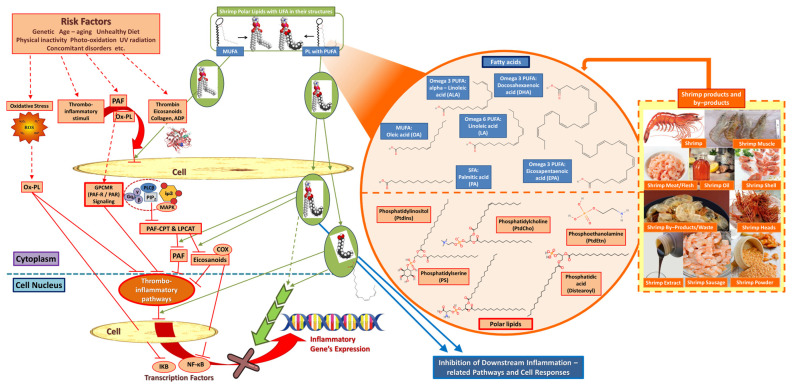
Anti-inflammatory properties of shrimp lipid bioactives (polar lipids and their MUFAs and PUFAs). Shrimp PL beneficially affect specific inflammatory signaling (induced by the presence of several risk factors), by either affecting the binding of thrombo-inflammatory mediators like platelet activating factor and thrombin in their membrane receptors, and thus by inhibiting the associated signaling, or by modulating the metabolism of these inflammatory and thrombotic mediators towards reducing their levels, or through the interaction of their MUFA and PUFA content with the eicosanoids’ pathways towards reduction and/or resolution of inflammation. All these effects of shrimp lipid bioactives with anti-inflammatory properties also beneficially affect the expression of thrombo-inflammation associated genes (reduction) and simultaneously the expression of anti-inflammatory genes (induction) through NF-κB signaling.

**Figure 3:**
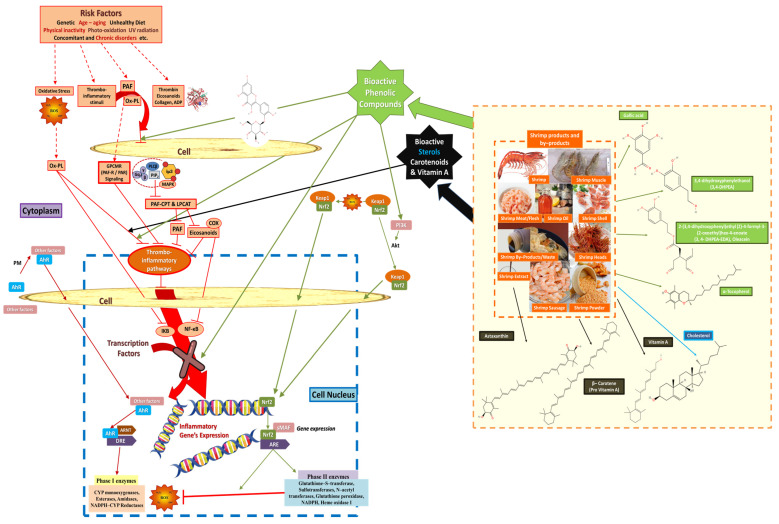
Anti-oxidant properties of shrimp carotenoids and amphiphilic phenolics. Shrimp carotenoids and amphiphilic phenolics have shown the capacity to beneficially scavenge the excess of free radicals produced during oxidative stress and inflammatory manifestations induced by the presence of several risk factors, and thus provide antioxidant protection of cell membranes, membrane polar lipids, MUFAs and PUFAs, and associated cell–cell interactions. Moreover, shrimp phenolic bioactives also affect specific inflammatory signaling by either affecting the binding of thrombo-inflammatory mediators like platelet activating factor and thrombin in their membrane receptors, and thus by inhibiting the associated signaling, or by modulating the metabolism of these inflammatory and thrombotic mediators towards reducing their levels. Shrimp phenolics and carotenoids have also been shown to beneficially affect the expression of oxidative-stress-associated genes (reduction) and simultaneously the expression of anti-oxidant genes (induction), through NRF2 signaling.

Lipids have an important role in keeping the cardiovascular system balanced and healthy, but they can also disrupt its harmonious functionality easily when consumed in larger amounts. Shrimp contain many beneficial lipids, especially FAs, and more specifically n-3 PUFA, which are important regulators towards blood pressure, energy, inflammation, etc., while ALA, DHA, and EPA are beneficial lipids necessary for living organisms that cannot produce them autonomously and have to consume them via our diet. Moreover, polar lipid bioactives, especially those that bear n-3 PUFA within their structures, have also been proposed as important anti-inflammatory dietary lipids with cardioprotective properties through specific mechanistic effects on thrombo-inflammatory signaling and cell–cell interactions (Figure 2). Moreover, shrimp also contain many carotenoids, especially astaxanthin, and marine phenolics known for their antioxidant and anti-inflammatory properties [57] against specific cellular mechanisms of oxidative stress and inflammation (Figure 3). Another benefit stemming from consuming shrimp is that their by-products also contain high amounts of lipids and antioxidants, meaning that they are adequate sources of health-promoting compounds suitable for food supplements, and even marine drugs. Interestingly, research has shown that shrimp extracts, as well as their lipids, carotenoids, and many other biomolecules, are advantageous towards the cardiovascular system, as shown by several studies presented in Table 5.

### 3.1. Anti-Inflammatory Action of Shrimp Extracts

Shrimp play a vital role in the aquaculture and marine food industry, due to their beneficial composition and their nutritional value. Shrimp PLs have been pointed out as major contributors in signaling, lowering the percentage of blood lipids, and protecting the cardiovascular system in general. It has been proven that dietary PLs promote anti-inflammatory action against chronic diseases as well [86,87]. Moreover, astaxanthin regulates various target genes related to inflammation, including inflammatory mediators like the interleukin 1 beta (IL-1β), tumor necrosis factor alpha (TNF-α), and interleukin 6 (IL-6), and participates in the blockage of pro-inflammatory signaling pathways, like the nuclear factor kappa-light-chain-enhancer of activated B cells (NF-κB), c-Jun N-terminal kinases (JNK), and the nuclear factor erythroid 2-related factor 2 (Nrf2) biochemical pathway [88].

#### 3.1.1. In Vivo Anti-Inflammatory Action

In vivo studies conducted on zebrafish proved the anti-inflammatory action of shrimp lipids. Generally, the role of macrophages is to detect and destroy any potential pathogens that could promote harmful effects on the organism. However, when macrophages are exposed to inflammation, they release cytokines, such as TNF-α, IL-1, IL-6, IL-8, and IL-12, which are known for their pro-inflammatory action. Further, macrophages release molecules with pro-inflammatory activity, such as prostaglandins like prostaglandin E2 (PGE-2), leukotrienes, and chemokines [89]. Several studies on zebrafish *larvae* models indicated the anti-inflammatory action against inflammation induced by CuSO_4_. This reagent can increase the expression of various inflammatory genes such as COX-2, TNF-α, and IL-1 [90]. A research pattern focused on the quantification of inflammatory cells, and more specifically macrophages, that migrated on zebrafish embryos’ tails. The collected findings showed that shrimp head lipid extracts, when compared to the CuSO_4_ group, led to a reduction of migrated macrophages [91,92,93]. Moreover, in order to be certain about the anti-inflammatory effects of shrimp lipid extracts, the tail-cutting of zebrafish *larvae* shrimp was chosen. The tail-cutting group exhibited obvious red macrophage aggregation, while on the contrary red macrophage migration of phospholipid extracts from shrimp heads decreased significantly at higher concentrations. This reduction of macrophages compared to the control group revealed an anti-inflammatory capability of shrimp polar lipids [92].

#### 3.1.2. In Vitro Anti-Inflammatory Action

An in vitro study in blood samples of almost 100 people of different sexes and races was held to evaluate the reduction of specific pro-inflammatory lipid mediators from n-3 FA supplementation. A group of participants were fed with marine-derived n-3 FAs daily, specifically 460 mg EPA and 380 mg DHA, and displayed a reduction in proinflammatory mediators, including prostaglandin D2 (PGD2) and 5- and 12-hydroxyeicosatetraenoic acid (5-HETE and 12-HETE) (−40.34%, −14.59%, and −22.28% respectively), within a year [94]. Apart from the evaluation of the proinflammatory mediators’ changes, it is also important to assess other indicators, such as high values of nitric oxide (NO), which can be easily quantified using in vitro methods. Abnormal levels of NO originate from the dysfunction of inducible nitric oxide synthase (iNOS), especially in cardiovascular diseases [95]. A study based on the quantification methods of NO used the RAW 264.7 cell line to calculate the anti-inflammatory ability of shrimp lipids extracted from the cephalothorax, which consisted of OA, PA, LA, DHA, EPA, stearic acid (SA), and ARA in both an encapsulated and non-encapsulated form. This cell line produces NO as a response to induced stress, while the anti-inflammatory property has an inversely proportional relationship with the amount of NO produced, i.e., the lower the production of NO, the higher the anti-inflammatory activity of the given product, as in this case considering the lipid mixture extracted from *L. vannamei* shrimp cephalothorax. Indeed, the anti-inflammatory effects were extravagant. When these cells were in contact with the encapsulated form of the lipids (100 μg/mL), the NO amount produced dropped from 42.42 μΜ to 27.51 μΜ, proving the powerful anti-inflammatory action of the shrimp’s lipids. Concurrently, when they were in contact with lower amounts of lipids, there was still a reduction in the NO amounts, albeit lower. However, it was noticed that when in contact with the non-encapsulated form of the lipids, there was no specific change, a fact attributed to low water solubility as the sample’s cells were not exposed enough to water in order to produce results [96]. All things considered, shrimp lipids and astaxanthin extracts can be used as supplements, or may even be included in medical drugs so as to promote vascular health and to prevent inflammation.

### 3.2. Antioxidant Action of Shrimp Extracts

Oxidative stress is one of the main contributors that lead to cardiovascular diseases. It is caused by high levels of reactive oxygen species (ROS) in the body and low antioxidant levels. ROS include superoxide anion, singlet oxygen, hydroxyl radical, hydrogen peroxide, and hypochlorite. In low concentrations, ROS are important for signaling, gene expression, cell growth, migration, and differentiation [97]. On the other hand, when ROS levels are higher than normal, oxidative stress creates an imbalance in blood circulation and cardiovascular diseases [98]. Hence, it is important to seek out natural biomolecules that can scavenge all the above-mentioned radicals.

Astaxanthin is a strong antioxidant carotenoid, due to its special structure, which is able to scavenge radicals in lipophilic and hydrophilic environments [99,100]. 2,2-diphenyl-1-picryl-hydrazyl (DPPH) is a method used to evaluate the antioxidant capability. A study using this method measured the astaxanthin DPPH-scavenging activity with and without the presence of liposomes. All liposomes that were loaded with 0.8 g of astaxanthin had high DPPH activity (81.76%). It is also important to note that liposomes which consisted of 70% phosphatidylcholine (PC), with a phospholipid concentration of 2 g/100 mL, showed the highest residual DPPH-scavenging activity. Furthermore, the chelating effect of ferrous metal was evaluated in the same research to test the antioxidants. It was found that the maximum chelating activity was exhibited by astaxanthin-loaded liposomes that contained 70% PC with a phospholipid concentration of 2 g/100 mL, throughout the storage period [101].

In another relative study, various methods were used to determine the antioxidant activity of extracted oil from shrimp containing astaxanthin. Namely, assays including the total antioxidant capacity (TAC), reducing power, hydroxyl radical sequestration (OH), DPPH radical sequestration activity, and oxygen radical absorbance capacity (ORAC), were used. Two different oils were extracted from the shrimp, apart from soybean oil which was utilized as the control sample: dry pigmented oil (WO) and pigmented oil of the shrimp residue meal (MO). WO samples showed greater antioxidant activity in some tests (TAC, reducing power, hydroxyl radical), meaning that they had lower values than MO, due to the higher astaxanthin concentration in the oil. During the experiment, it was also noticed that when heat was applied, astaxanthin extraction was better, meaning that astaxanthin can be recovered to the maximum possible yield while cooking [102].

In addition, experiments on RAW Cell 264.7 cell lines were conducted with encapsulated and non-encapsulated shrimp lipid extracts. A photochemiluminescense assay was validated to measure the antioxidant activity of shrimp lipids extracted from the cephalothorax. More particularly, this method involves the detection of luminol, which acts as an oxygen radical detection reagent. To evaluate the antioxidant activity in lipophilic and hydrophilic environments, the lipid-soluble (ACL) and water-soluble antioxidant capacity (ACW) were used, respectively. Comparing non-encapsulated and encapsulated lipid extracts, significant differences were traced in the antioxidant activity. Specifically, non-encapsulated lipid extracts did not show any antioxidant activity (ACL: not detected, ACW: not detected), compared to the encapsulated lipid extracts from shrimp (LES) (ACL: 1.692 ± 0.381 ng Trolox/μg LES, ACW: 14.853 ± 0.465 ng ascorbic acid/μg LES) [96]. A similar in vitro study proved that astaxanthin extracted from shrimp by-products has high anti-oxidant activity in non-toxic quantities due to its structure which traps free radicals, making them unable to act in an oxidative manner. The experiment was conducted on HS68 cells and cell viability was able to be controlled. Astaxanthin, provided at an amount of 0.2 nM, altered the viability protection from 59.10% to 83.57%. Through this experiment, this natural antioxidant seemed to have the same antioxidant capacity as the synthetic antioxidant N-acetyl cysteine (NAC), confirming its importance and its ability to be easily indexed in shrimp [103]. The antioxidant action of shrimp was also tested in the food industry on shrimp sausages, and on the third day of fermentation, shrimp sausage exhibited an antioxidant capacity of 42.09% [104].

The antioxidant activity was evaluated by research in vivo, using zebrafish larvae models. Metronidazole (MTZ) was administrated to zebrafish, which caused ROS overproduction, apoptosis of skin cells and reduction of fluorescent spots (FS). It was observed that among the different sources of lipid oils, the shrimp head lipids presented the highest antioxidant ability. More specifically, the extracts consisted mostly of glycerophospholipids and triacylglycerols, such as PC(18:1(11Z)/20:5(5Z,8Z,11Z,14Z,17Z)), PC(16:0/22:6(4E,7E,10E,13E,16E,19E)) and TG(16:0/18:0/18:2(9Z,12Z)), respectively, according to the UPLC-Q-Exactive Orbitrap/MS qualitative analysis [93]. The reportedly great antioxidant activity of shrimp could be a cheap and resourceful measure for the prevention of many pestering diseases that are induced by oxidative stress.

### 3.3. Antithrombotic Action of Shrimp Extracts

Generally, marine animals’ PLs have strong antithrombotic activity against the platelet-activating factor (PAF) and thrombin pathways [105]. The antithrombotic effects can be linked to inflammation and oxidative stress, meaning that lower inflammation is followed by a lower chance of thrombosis [106]. Different studies, using zebrafish models to evaluate the shrimp lipid extracts’ antithrombotic activity, measured the fluctuation of the staining intensity values, a quantification method aiming to calculate the concentration of erythrocytes.

High levels of SI of erythrocytes are an indication of thrombosis. All gathered studies agreed that the shrimp lipid groups’ SI of erythrocytes reduced to a smaller degree, in contrast to the control ones [91,92,93]. Moreover, many researchers proceeded to evaluate the antithrombotic effects through the thrombogenicity index, which shows the tendency of thrombogenicity and, more specifically, the relation between pro-thrombogenic (SFAs) and anti-thrombogenic FAs (UFAs) [107]. All results indicated that the thrombogenic index of lipids obtained from flesh displayed a briefer range compared to the one of lipids from shrimp waste (0.17–0.36 and 0.18–0.46 respectively) [23,38,48,72,108]. Thus, this indicator of nutritional quality is influenced not only by the season of the measurement, but also by the location of shrimp species [109]. Furthermore, non-berried females and berried females had significant differences in the values of this index, as well as post-moon and pre-moon shrimp [75]. Additionally, it was observed from another research that shrimp oil extracted from hepatopancreas (HPO) showed a significantly lower thrombogenicity index than the oil from cephalothorax (CPO). This may be attributed to the higher amount of n-3 FAs reported in HPO [49]. There is no specific recommendation for this index; however, lower values are correlated with preventing cardiovascular problems and platelet aggregation. This can be explained further by the drawbacks SFAs provide when consumed in larger amounts, and also by their less valuable action in comparison to PUFAs and MUFAs. SFAs are part of the hypercholesterolemic group of FAs, and through the reduction of low-density lipoproteins’ action, they increase blood cholesterol levels, leading to inflammation and thrombosis. Keeping the hypercholesterolemic factor lower than the hypocholesterolemic one, the antithrombotic action is enhanced, as shown in the indexes of shrimp species that, in contrast to other marine animals, were found at lower values, proving the unique benefits of shrimp.

The index of thrombogenicity used in the studies refers to the ratio of thrombus formation FAs, while the fact that FAs have antithrombotic activity can be calculated by the following equation:

(IT) = [C14: 0 + C16: 0 + C18: 0]/[(0.5 × Σ MUFA) + (0.5 × Σ n-6 PUFA) + (3 × Σ n-3 PUFA) + (n-3/n-6)](1)

### 3.4. Anti-Atherogenic Action of Shrimp Extracts

The term atherosclerosis refers to the build-up of plaque inside the arteries, which consists of fats, cholesterol, and other substances. The accumulation of plaque leads to constriction of the arteries, which implies thrombosis [110]. Studies show that shrimp flesh and waste extracts provide anti-atherogenic action, measured by the atherogenicity index. The range of this nutritional quality indicator was found in flesh extractions ranging from 0.19 to 0.53 and in shrimp waste from 0.25 to 0.42 [23,48,49,72,108,109]. Moreover, this index was affected by the season, due to the different recorded lipid compositions of shrimp. Values under one indicate that the action of MUFAs and PUFAs is more prevalent than the action of SFAs. Consequently, our cardiovascular system is highly protected against atherogenicity [38]. Another study that was conducted on the *P. semilaevis* genus in different periods (pre- and post-monsoon) revealed that the atherogenicity index was calculated within the range of 0.48–0.62 for pre-monsoon and 0.22–0.3 for post-monsoon shrimp, but their sex did not seem to affect the outcome. This index is important for a better understanding of the metabolic effect of FAs composition in shrimp. Low index values indicate that shrimp possess higher amounts of PUFAs, which is linked to a nutritious diet able to aid in the reduction of cardiovascular diseases [75].

The atherogenicity index used in the studies refers to the ratio of proatherogenic SFAs (myristic acid, stearic acid, lauric acid) and anti-atherogenic activity of UFAs, and can be evaluated by the following equation:

(IA) = [C12: 0 + (4 × C14: 0) + C16: 0]/[Σ MUFAs + Σ PUFAn6 + PUFAn3](2)

### 3.5. Proangiogenic Action of Shrimp Extracts

Angiogenesis is a biological process that includes the formation of new blood vessels. This process is important for the effectiveness of wound healing and is responsible for supplying organs and tissues with oxygenated blood [111,112]. A tyrosine kinase inhibitor, namely PTK787, may initiate vascular damage and enable angiogenesis in zebrafish embryos. In both evaluated studies that used this method, all the shrimp lipid groups exhibited vascular growth-promoting effects in comparison to the PTK787 group, which corresponded to proangiogenic activity. In addition, the majority of measurements indicated a dose-dependent correlation between lipids and the proangiogenic activity, with only one exceptional outcome that needs to be further analyzed [91,92].

### 3.6. Shrimp Lipids on Hypercholesterolemia

Hypercholesterolemia is a condition associated with high levels of low-density lipoprotein cholesterol (LDL) in the blood. High LDL values affect human health and are responsible for the onset of cardiovascular diseases (CVDs) [113]. Recent studies used the hypocholesterolemia/hypercholesterolemia index (h/H index) to evaluate the nutritional value and health benefits of the consumption of shrimp. The h/H index refers to the ratio of hypocholesterolemic and hypercholesterolemic FAs and the effects of specific FAs on cholesterol’s metabolism. The recommended value for the h/H index is >1.0. There have not been enough studies using this index for evaluation, but the range fluctuates from 1.52 to 3.10 in shrimp meat or homogenized mixtures and approximately from 2.40 to 2.55 in shrimp by-products. Thus, all studied shrimp are within the recommended values and can be considered a good anti-hypercholesterolemic source [38,48,49].

### 3.7. Shrimp Lipids on the Protection of the Endothelial Cells

Endothelial cells play a major role in the balance and operation of the cardiovascular system. Their main function lies upon the formation of a thin layer that covers the interior surface of all blood and lymphatic vessels, while their main role is to regulate blood pressure and flow, to control substance trafficking through and out of the blood, and to maintain tissue homeostasis. Vascular endothelial cell chronic malfunction may lead to cardiac arrest and stroke due to the induced imbalance [114,115]. Hence, maintaining the health of endothelial cells is an important task that requires developing research, starting with their endoplasmic reticulum. Endoplasmic reticulum (ER) stress may arise by the accretion of unfolded proteins, general oxidative stress, and calcium imbalance [116], which cause the dysfunction of endothelial cells and lead to unwanted effects. Three regulatory pathways deal with the created stress: the protein kinase RNA-like ER kinase (PERK), inositol requiring protein-1 (IRE-1), and the activating transcription factor-6 (ATF6), all responding to ER stress which is controlled via chain reactions [117].

Since shrimp have gained growing attention in the marine drugs domain, shrimp lipids were tested to evaluate their protective effects on the ER stress mechanisms of endothelial cells. The samples were extracted from waste-considered shrimp remains (heads and tails) of *Litopenaeus vannamei* shrimp and were tested on EA.hy926 endothelial cells from the human umbilical vein cell line with various concentrations, which were assessed before so as to make sure that the cells would not inhibit the apoptosis and necrosis. To evaluate ER stress on the cells, the concentrations of ER stress-inducers thapsigargin (Tg) and tunicamycin (Tu) were analyzed. Cells that were only treated with these inducers inhibited cell viability to 44.97 ± 1% after Tg treatment, while after the treatment with shrimp lipids as well, cell viability increased to 72.42–77.36%. Regarding the Tu inducer, cell viability arose from 46.43 ± 2.06% to 67.31–69.09%, depending on the concentration of the lipid extract. Meanwhile, the percentage of apoptosis and necrosis was also evaluated so as to test whether the pretreatment with shrimp lipids would prevent apoptosis and necrosis on endothelial cells. Indeed, cells treated with Tg and Tu mediated the apoptosis from 41 ± 2.35% and 43.67 ± 2.6% to 10.72–12.22% and 15.94–16.61%, respectively, proving that pretreating endothelial cells with shrimp lipids may strengthen their defensive action.

Glucose-regulated protein 78 (GRP78) is also a very important regulator that may help against ER stress, because it speeds up the protein folding process and correspondingly decreases the chance of ER stress occurring [118]. Through Western blot analysis, it was found that Tg and Tu treatment increased the level of GRP78, while shrimp lipids reduced it. Immunofluorescence analysis was also used, and all results indicated that Tg treatment increased the signal of GRP78 while at the same time shrimp lipids decreased it. Cells pretreated with shrimp lipids before Tg treatment inhibited a reduced GRP78-positive signal compared to the outcomes provided via Tg treatment alone. The PERK protein regulator was also analyzed, due to its relation with ER stress and cell death. Cells pretreated with shrimp lipids before exposure to Tg, inhibited a defense against the increased phosphorylated PERK induced by Tg, and concurrently reduced the C/EBR homologous (CHOP) protein in the cells treated with Tg. Lastly, the level of cleaved poly (ADP-ribose) polymerase (PARP) needed to be evaluated. Cells treated with Tg demonstrated high amounts of cleaved PARP, while cells pretreated with shrimp lipids before Tg exposure inhibited the protection of the PRAP protein, confirming that shrimp lipids can block the apoptotic cell death caused by ER stress [119]. It was indeed proved that shrimp lipid extracts protect the endothelial cells and the cardiovascular system as well, which highlights the need for the introduction of shrimp species in our dietary pattern.

### 3.8. Shrimp Lipids on Mortality Due to Cardiovascular Diseases

Coronary heart disease is a very common reason for death among people, and its fatal form is known as fatal coronary heart disease (CHD). A major indicator of this disease is when the heart arteries are unable to deliver the necessary oxygen to the heart leading subsequently to death [120]. n-3 FAs are known for their beneficial role on the cardiovascular system; therefore, hundreds of participants during a test were fed with marine animals including shrimp and other ALA sources (soybean oil, walnuts, olive oil etc.) in order to assess the effects of these specific FAs on the mortality rates due to CVDs. After 5.9 years of a specific intake of FAs, 431 deaths were noted, including 55 cases of fatal CHD, 32 cases of Sickle Cell Disease (SCD), and 25 cases of fatal stroke. However, participants consuming at least 500 mg/day of EPA and DHA, constituents that are metabolized with the help of ALA, inhibited a reduction of SCD risk by 52%, as well as CVDs and CHD by 39% and 46%, respectively. An ALA intake of 0.7% for the participants led to reduced mortality rates by 28% [121]. Overall, the importance of n-3 FAs and their benefits were thoroughly analyzed, and it was proved that mortality rates due to CVDs could be effectively reduced by sticking to a balanced diet that contains the necessary amounts of n-3 FAs.

**Table 5:** Studies, interventions, and clinical trials on the benefits of bioactive lipid extracts derived from shrimp against inflammation, thrombosis, and cardiovascular diseases.

Hypothesis/Intervention	Study Design/Parameters Examined	Main/Concluding Observed Benefit(s)	Specific Benefits/Other Benefits/Mechanism of Action	Year	Ref.
This study aimed to evaluate zebrafish model shrimp head lipids for their pro-angiogenic, anti-inflammatory, antithrombotic, and cardioprotective activities.	A zebrafish model was used to evaluate all pre-mentioned properties of shrimp head lipids.	Each group’s lipids demonstrated pro-angiogenic, anti-inflammatory, and cardioprotective activities. Some groups also displayed specific antithrombotic activities.	All shrimp head lipids reduced macrophage migration. Zebrafish in the model group (80 μM ARA), induced increased caudal vein thrombosis. Heart injury symptoms appeared in this group, mitigated by the positive control and lipids from four species.	2022	[91]
This study aimed to initially compare the activity differences among zebrafish model extracts and evaluated the in vivo effects of phospholipids from three materials.	Zebrafish (*Danio rerio*) abdominal muscle (AB) strains were utilized so as to study the angiogenic, inflammatory, cardioprotective, and antithrombotic activities of this shrimp species.	Phospholipids from different sources varied in antithrombotic, anti-stroke, anti-inflammatory, proangiogenic, and cardioprotective activities, with shrimp head’s phospholipids (SH) notably promoting vascular growth.	Codfish roe (CR), SH, and squid gonad (SG)-derived phospholipids demonstrated significant antithrombotic and anti-inflammatory effects, with SH specifically enhancing the cardioprotective activity in zebrafish.	2022	[92]
This study evaluated the antithrombotic, antioxidant, and anti-inflammatory activities of lipid extracts from three materials using the zebrafish model in vivo.	Zebrafish (*Danio rerio*) and AB strains were used so as to estimate their antioxidative, anti-inflammatory, and antithrombotic activity in vivo.	All lipid groups showed strong antioxidant and anti-inflammatory activities. All SH lipid groups and one soybean lipid group (90 μg/mL), showed an obvious antithrombotic activity as well.	In 99 differential lipids between soybean and SH extracts, glycerophospholipids (GPs) correlated positively with antithrombotic and antioxidant activities, while glycerolipids (GLs) correlated positively with anti-inflammatory activity and negatively with the other two activities.	2020	[93]
This study evaluated the bioactive and technological functionalities of shrimp waste lipid extract.	LES was spray-dried into a water-soluble powder and tested for its anti-inflammatory and antioxidant activities.	Shrimp waste lipid extract is promising for food due to composition, anti-inflammatory, antioxidant activities, coloring, and stability.	Spray-drying encapsulation notably enhanced shrimp waste lipid extract’s water solubility, as well as its antioxidant, anti-inflammatory activities, and astaxanthin bio-accessibility to 100%.	2018	[96]
This study aimed to explore shrimp oil effects from shrimp waste on ER stress in human endothelial cells.	Human endothelial cells were pretreated with shrimp lipids (250 and 500 μg/mL) before Thapsigargin exposure.	Shrimp lipids reduce ER stress in endothelial cells, which ispromising for vascular disease prevention and treatment.	Shrimp lipids suppressed the ER stress-regulator GRP78 and attenuated PERK and IRE-1 pathways, inhibiting ER-related apoptosis.	2022	[119]
The effects of two phospholipid compositions and concentrations on astaxanthin-loaded liposome properties were compared.	The cellular uptake of astaxanthin-loaded liposomes by Caco-2 cells was investigated to assess astaxanthin bioavailability.	Our data indicate that astaxanthin bioavailability in liposomes depends on PC properties and liposome size.	Liposomes with 70% PC (2% *w*/*v*) had a higher astaxanthin entrapment, uptake, and antioxidant activity relative to 23% PC.	2018	[101]
Extracted astaxanthin-rich materials from shrimp (*L. vannamei*) industrial residue and meal were evaluated for their physicochemical characteristics and antioxidant potential.	Evaluated TAC, reducing power, OH radical scavenging, DPPH scavenging, and ORAC for effectiveness.	Pigmented oils from shrimp residues showed great potential concerning the food industry, due to affordability and high antioxidant activity.	The smaller-scale residue meal (MO) had significantly higher antioxidant activity, due to its concentrated astaxanthin, and thus holds great health benefits.	2021	[102]
The antioxidant activity, vitamin E levels, LAB, pathogenic bacteria, acidity, and acceptance in fermented shrimp sausage were studied.	An experiment with 0, 1, 2, and 3 days of fermentation, was used so as to measure the antioxidant activity using the ABTS radical method.	The best formulation was obtained from the first-day fermented shrimp sausage, scoring 2.49 in hedonic tests with 26.57% antioxidant activity.	Significant antioxidant activity and vitamin E content were found in first-day fermented shrimp sausages.	2022	[104]
This study highlights the nutritional benefits of deep-water shrimp for human diets.	Shrimp’s thrombogenic and atherogenic effects, were analyzed using atherogenic (AI) and thrombogenic (TI) indexes.	Lower thrombogenicity and atherogenicity indices suggest deep-water shrimp are valuable health food items for cardio-protection and anti-platelet aggregation.	-	2022	[108]
This study aimed to provide basic nutritional information on *M. nipponense* from the Anzali wetland.	Nutritional lipid quality was assessed using the AI and TI indexes.	Diets with the lowest amounts of AI and TI might decrease the potential risk of CHD.	The indexes of nutritional quality of lipids (AI and TI) presented low values, representing the beneficial health effect of these invasive species.	2018	[109]
This study aimed to compare oils from Pacific white shrimp’s cephalothorax (CPO) and hepatopancreas (HPO).	The comparison was assessed with the TI and AI indices and the h/H ratio.	Based on nutrition indices, HPO offers more health benefits than CPO, due to the higher PUFA content and increased h/H ratio.	A similar AI index was observed between CPO and HPO (*p* > 0.05). However, TI was lower in HPO, compared to that of CPO. HPO possessed a higher h/H ratio and PUFA/SFA ratio.	2021	[49]
This study aimed to highlight the shrimp’s nutritional value and the potential deriving from utilizing shrimp processed waste.	The FA profile determined nutritional parameters: PUFA/SFA, PUFA/MUFA ratios, and h/H fatty acids. Additionally, AI and TI indexes were calculated.	All findings implied many health benefits following the consumption of fat from red shrimp.	High PUFA/SFA and h/H fatty acid ratios, along with low AI and TI values, were observed.	2021	[19]
This study aimed to shed a light on the potential thrombogenic and atherogenic impacts of the consumable portions of shrimp.	The TI and AI indexes were assessed according to Ulbricht and Southgate (1991).	A higher PUFA content with lower TI and AI indexes indicates the potential of shrimp to promote cardiovascular health.	AI ranged from 0.48 to 0.62 (pre-monsoon) and 0.46 to 0.63 (post-monsoon). TI ranged from 0.22 to 0.3 (pre-monsoon) and 0.32 to 0.42 (post-monsoon).	2024	[75]
In this study it was hypothesized that low-fish-intake participants would show significant eicosanoid changes post-marine n-3 supplementation.	VITAL was a double-blind trial with 25,871 participants studying vitamin D3 and marine n-3 effects.	n-3 supplementation for 1 year showed favorable changes in mediators, more pronounced in low-fish-intake groups.	After 1 year of n-3 supplementation, a reduction in proinflammatory specialized pro-resolving mediators (SPMs) (PGD2, 5-HETE, 12-HETE) and an increase in pro-resolving mediators (15-HETE, EPA, and DHA), as well as resolvins D1 and D4 (RvD1 and RvD4, respectively)), were observed compared to placebo.	2024	[94]
This work aimed to evaluate extracts from pink shrimp residue for their anti-obesity and mixed hypolipidemic properties.	This in vivo study on Swiss Mus musculus mice, evaluated the effects of shrimp extracts on weight, cholesterol, triacylglycerols, and glucose levels during a 30-day high-fat diet.	Shrimp extracts reduced cholesterol and triacylglycerol levels and led to weight reduction in mice.	These effects are probably due to a synergistic action of astaxanthin, EPA, and DHA FAs.	2015	[122]
In this study it was hypothesized that ALA intake prevents fatal CVDs and all-cause mortality, alongside high fish-derived LCn-3PUFA intake.	A randomized clinical trial was conducted in a population fed with high fish consumption and dietary ALA and marine n-3 fatty acids.	Fish-derived EPA and DHA promoted protection from cardiovascular and cardiac death.	Marine and vegetable n-3 FAs act synergistically and are partners rather than competitors in reducing mortality.	2015	[121]
This study aimed to evaluate lipid nutritional quality indices of two shrimp species collected in Santa Catarina, Brazil.	The lipid fraction’s nutritional quality was assessed using the TI and AI indexes, as well as the h/H ratio.	Nutritional quality indexes suggested potential health benefits from both studied shrimp species.	Pink shrimp had AT and TI values of 0.27 and 0.21, respectively, with h/H > 2.0. Pacific white shrimp had AI and TI values of 0.19 and 0.26, respectively, also with h/H > 2.0, indicating superior FA composition.	2018	[38]
Nutritional indices and ratios were assessed regarding the tested species’ nutritional quality and health benefits.	Fish and shellfish tissues were evaluated using nutritional indexes: AI, TI (Ulbricht and Southgate), and h/H ratio (Santos-Silva et al.).	These species were also promising and valuable natural sources of essential biologically active FAs, besides the Black Sea fish.	Lower lipid indices (AI < 1, TI < 1) and higher h/H ratios (0.8–1.78 for fish, 1.52–4.67 for bivalves and shrimp) indicated health benefits from Black Sea fish and shellfish.	2021	[48]

## 4. Other Health Benefits and Effects of Shrimp Extracts Rich in Lipid Bioactives

Shrimp extracts are claimed to have an excellent influence on the health of the cardiovascular system, due to their lipid composition and especially the beneficial PUFAs combined with lower amounts of SFAs and due to their high concentration of carotenoids which are strong antioxidant and anti-inflammatory contributors. However, studies have shown that shrimp extracts affect other cell lines and can be useful for improving the condition of those suffering from neurological diseases, cancer, chronic illnesses, and many others. This section is dedicated to a series of several other health benefits attributed to shrimp extracts and how such extracts can be used in several applications for the prevention of inflammation and oxidative stress related chronic diseases; the studies are summarized in Table 6.

### 4.1. Anticancer Effect of Shrimp Extracts

Cancer is one of the most common deadly diseases, and researchers globally have sought to discover the best possible cure. Many chemotherapeutic molecules are currently in use; however, they contain many side effects and many cancers are drug-resistant. This has led to new research that has been conducted in recent years by many researchers into natural anticancer biomolecules. Marine animals seem to contain such biomolecules, particularly shrimp. Research has shown that shrimp may have anticancer action via their lipid compounds, especially carotenoids, which are linked to apoptotic investiture and oxidative stress promotion of cancer cells [123]. Extracts from *L. vannamei* were tested on five different cancerous human cell lines (A-549 (lung carcinoma), HCT116 (colon carcinoma), HeLa (epithelioid cervix carcinoma), MDA-MB-231 (breast adenocarcinoma), and 22Rv1 (prostate carcinoma)) and one non-cancerous (ARPE-19 (retinal pigmented epithelium)). The antiproliferative activity (prevention of proliferation of cancerous cells) was tested using the MTT assay and the selection of the most bioactive fractions was based on their half-maximal growth inhibition (GI50) values; lower GI50 values indicated higher bioactivity of the tested fractions.

An extract using methanol solvent (M11) showed antiproliferative activity on human cell line 22Rv1 (prostate cancer), with GI50 values of 70.6 ± 5.8, 71.0 ± 4.5, and 70.3 ± 5.6 μg/mL, and with these promising results it was further fractionated for analysis. The sub-fraction M.11.B stood out, since it was the most bioactive fraction against the 22RV1 cell line, with GI50 values of 46.6 ± 2.2 μg/mL, indicating that the fraction increased the antiproliferative activity since the GI50 value decreased from 70.6 ± 5.8 (in M.11) to 46.6 ± 2.2 μg/mL (in M.11.B), confirming its effectiveness towards these specific cancerous cells. Through extensive analysis, it was observed that the fraction consisted of different compounds, including EPA, proving that shrimp extracts may be used as anticancerous biomolecules, since EPA is one of the most common FAs found in large amounts in shrimp and it can prevent cancer cell proliferation by modifying the expression of genes involved in the cell cycle [124].

Another study was conducted on the lipid composition of many marine animals, including Argentina red shrimp (*Pleoticus muelleri*), that focused specifically on their potential anticancer properties. The lipids were tested on the HCT116, A2058 (Melanoma), A549, T98G (Glioblastoma multiforme), and HeLa (cervix adenocarcinoma) cell lines, and the cytotoxicity effects were analyzed with Microplate Spectrophotometer utilizing the IC_50_ concentration. Results were very promising as Argentina red shrimp’s lipid extracts had the biggest cytotoxic effect on all cancer cells investigated. This was noticed in the results of the spectrometry through the IC_50_ concentration for HCT116, A2058, A549, T98G, and HeLa of 27.2, 29.0, 14.9, 12.3, and 33.9 μg/mL, respectively, while the rest of the marine animals exhibited higher concentrations, which indicated their lower bioactivity. This in vitro analysis showed that shrimp had the biggest anticancer activity out of the eleven tested marine animals that are also highly consumed, indicating the shrimp’s important role in the human diet [72]. Similar research on shrimp extracts was conducted in vitro on A549, 22Rv-1, HeLa, MDA-MB-231, HTC116, and ARPE-19 cells that were used as control samples. Seven different fractions were obtained from open column chromatography with different concentrations, and the results were very promising and similar to other studies that proved the anticancer nation of the shrimp extracts. Two extracts (C3 and C5) of 200 g/mL were tested on 22Rv1 cells and exhibited great antiproliferative action through the reduction of the cell viability. These results were comparable to another study mentioned above, conducted with *L. vannamei* muscle extracts consisting of EPA and some non-lipid compounds with anticancer effects on the same cell line [124]. Lung adenocarcinoma cell line A549, which is used for one of the most common types of lung cancer with a high mortality rate, was also treated with a C5 fraction of 200 g/mL and the reduction of viability was again an indicator of its antiproliferative activity.

Similar effects to those mentioned above were noticed on HCT116 and MDA-MB-231 cell lines when they are treated with the fraction C5. All gathered research outcomes imply that an innovative discovery has been made, since breast cancer is also one of the most common cancer types with high mortality rates. However, no effects were noticed when the HeLa cell line was treated with the fractions, and no different results were recorded after ARPE-19 cell lines’ treatment as well, which may lead to the conclusion that these shrimp extracts possess specific selectivity towards cancerous cells [125]. The results were verified through another analysis using the same method, which concluded that triacylglycerols, PUFAs, and SFAs, especially EPA, possess strong antiproliferative properties on cancerous cells [20].

Carotenoids have also displayed significant anticancer properties. Astaxanthin, extracted from shrimp (*Penaeus Monodon*) to an amount of 50 ± 2 mg/g dry weight (DW), was tested on MCF-7 cells, where cell viability was noticed in 48 h (78 to 32%). The IC_50_ concentration was 19.8–20.2 μΜ. This experiment also pointed out that carotenoids from shrimp mixed with carotenoids from spinach may display even bigger results; however, further analysis is required [126]. Another study confirmed these results, using extracts from shrimp *Penaeus monodon* to test the effect of astaxanthins and other carotenoids such as β-carotene on cancer cells. The extracts were tested on H1975 (T790M mutation), H3255 (L858R mutation), and H441 (wild type) cell lines found in lung cancer patients. The extract had a great influence on the H1975 cell line with the IC_50_ reaching 2.77 μg/mL. If the IC_50_ is lower than 20 μg/mL, there is a high chance that the same results will be noticed in in vivo experiments as well [127]. Therefore, the anticancer action of shrimp’s lipid composition and carotenoids has been established through many in vitro and in vivo experiments, which imply their great promise towards cancer prevention.

### 4.2. Antidiabetic Effects of Shrimp Extracts

Diabetes is a chronic disease caused by cellular inability to produce or use insulin optimally. Diabetes is a general category, which includes type 1 diabetes mellitus (T1DM) and type 2 diabetes mellitus (T2DM). T1DM is considered an autoimmune disease caused by the destruction of pancreatic β-cells and entails low insulin and c-peptide levels in plasma. On the other hand, T2DM arises from insulin resistance and insulin deficiency, due to its’ insufficient signaling. Commonly, T2DM is not an autoimmune disease but is caused by inflammation, mitochondrial dysfunction, and amyloid deposition [128,129,130,131]. Leptin is a significant biomolecule for the regulation of glucose metabolism. Recent studies have shown that leptin deficiency causes severe insulin resistance, which leads to diabetes mellitus. Another biomolecule which is important for the regulation of glucose levels and takes part in the regulation of glucose metabolism is adiponectin. Abnormal adiponectin levels, either higher or lower, can induce several diseases, including diabetes mellitus [132]. Furthermore, glycated hemoglobin consists of carbohydrates, most commonly monosaccharides, linked with hemoglobin. The linkage with the sugar molecules occurs due to excessive sugar levels in the bloodstream.

The quantification of hemoglobin A1C (HbA1c), can be used for the diagnosis of diabetes mellitus. Diabetic rats were used in many studies, so as to examine the antidiabetic ability of shrimp lipids and astaxanthin extracts. The inclusion of shrimp oil in the rats’ diet, which consisted of high fats (control), showed a significant increase in blood glucose levels, compared to rats fed a low-fat diet (control) by oral glucose tolerance tests (OGTT). In parallel, serum insulin, leptin, and adiponectin were measured by ELISA, leptin, and adiponectin kits respectively. Higher values of insulin and leptin were observed as shrimp oil dietary replacement increased, in comparison to the high-fat control diet, while serum adiponectin and HbA1c values decreased. Insulin sensitivity was evaluated using the homeostasis model assessment of insulin resistance and it was observed that shrimp oil was responsible for improvements in insulin resistance and also leptin resistance, providing shrimp the ability to prevent and treat T2DM [133]. Another study based on diabetic rats investigated the hypoglycemic activity of astaxanthin extracted from shrimp shells, which is actively associated with diabetes. More specifically, OGGT pointed out that astaxanthin reduced the glucose concentration in plasma, compared to the rats of the diabetic control group, which showed higher values of glucose and insulin. In addition, astaxanthin presented lower values of insulin resistance compared to the control group [134]. Diabetic retinopathy is enhanced by high blood sugar levels due to diabetes. A clinical trial that investigated the effects of dietary marine n-3 FAs in preventing diabetic retinopathy exhibited promising outcomes. Out of 3482 participants, 75% who met the LCn-3 PUFA recommendation target (500 mg/day), were compared to those who did not fulfil the recommendation target. This comparison exhibited a correlation between the intake of dietary LCn-3 PUFA, with a decreased appearance risk of diabetic retinopathy [21].

### 4.3. Anti-Obesity Effect of Shrimp Extracts

Obesity, is a very common chronic disease known for the excessive fat deposits on the body that can lead to other diseases, as mentioned by the WHO. Chronic obesity can induce adipocyte imbalance, which in turn can increase the tissue mass and size, while also increasing the insulin resistance of the body leading to T2DM and other CVDs. This is due to bigger amounts of concentrated fat than those needed for a healthy body. It can also affect reproduction, bone structure, and may even enhance cancer genesis because of chronic inflammation and oxidative stress factors [135]. Shrimp however, have exhibited anti-obesity effects as well, a theory also partially confirmed by the effects they have on cardiovascular system health. Adipose tissue is in charge of energy storage, lipid metabolism, glucose uptake, and the inflammation factor, so when an imbalance of its function occurs, health problems may arise [136].

An in vitro study using extracts from *Pandalus borealis* containing FAs and astaxanthin proves the anti-obesity properties of shrimp. Cells were treated with shrimp oil and extract, as well as esterified astaxanthin extracted from shrimp. As a result, a decrease in fat accumulation on the cells occurred, while on the contrary, fish oil tested on the same cells showed an increase in fat accumulation, proving once again the importance of shrimp in comparison to other marine animals. This conclusion was based on the decreased mRNA expression of peroxisome proliferator-activated receptor (PPARγ) in the ET3-L1 adipocytes compared to the control group of cells. The same decrease was noticed in the mRNA expression of the sterol regulatory element binding protein 1c (SREBP-1c) in 3T3-L1 adipocytes, and in diacylglycerol acyltransferase-2 (DGAT-2); however, astaxanthin treatment did not seem to affect this specific expression. Lastly, a decrease was noticed in the mRNA expression of stearoyl-CoA desaturase-1 (SCD1) after treatment with the shrimp extracts, but no differences were observed in the expression of the fatty acid synthase (FASN) and the glucose transporter type 4 (GLUT4). Overall, treating 3T3-L1 cells with shrimp extracts containing n-3 PUFAs, phospholipids, and esterified astaxanthin showed a reduction in fat accumulation and a lower mRNA expression of genes linked directly to adipogenesis and lipogenesis, while fish oil treated on the same cells proved the opposite point, and hence confirmed the importance of a diet containing shrimp [19].

This hypothesis was later on validated through an in vivo experiment where two different extracts from pink shrimp, using two different methods, were treated on mice. The extracts contained high amounts of PUFAs (from 8% to 26.8%) including PA, LA, EPA, and DHA. EPA and DHA were the most present constituents in the extract containing 26.8% PUFAs (11.21% and 7.95% respectively). The extract with 26.8% PUFAs showed a weight reduction up to 20% during the 30 days of treatment, while the extract with 8% PUFAs displayed only a 4% decrease in weight. Since all mice had the same balanced diet except the extracts, this hypothesis was further confirmed. The cholesterol levels of mice treated with 26.8% PUFAs exhibited high reduction from 667 ± 42 g/dL to 390 ± 51, proving the hypolipidemic effect of the shrimp extracts. Triacylglycerol levels were also measured, where mice treated with 8% PUFAs showed a reduction from 160 ± 7 to 149 ± 46 and the level of triacylglycerols in mice treated with 26.8% PUFAs did not alter. As demonstrated, the extract containing 26.8% PUFAs has high anti-obesity and hypolipidemic action, due to its effects on the LDL receptors of the mice. The cholesterol reduction was attributed to the phenolic content of the shrimp extract, while the reduction in triacylglycerols was attributed to the astaxanthin content in the in vivo test [122]. In summary, the anti-obesity action of shrimp extracts was confirmed both theoretically and experimentally and it was noted that shrimp are a powerful source of health maintenance and the prevention of other serious chronic diseases caused by obesity.

### 4.4. Shrimp Extracts on Neuroprotection and Alzheimer

Amyloid-beta (Aβ), is a protein highly connected to oxidative stress and neuroinflammation. Excessive amounts of Aβ, can lead to the production of ROS and pro-inflammatory cytokines that enhance neural degeneration and chronic diseases such as Alzheimer’s disease, a widely known disease for its catastrophic effects on the neural system [137]. However, n-3 PUFAs may act as a solution against Alzheimer’s and other neurodegenerative diseases, due to their anti-inflammatory and antioxidant action. OA is responsible for controlling mitochondrial dysfunction, insulin resistance, and inflammatory signaling, and it can act as a neurotrophic agent. Palmitoleic acid, on the other hand, is also known for its neuroprotective nature [138].

Shrimp are known for their rich lipid composition, are considered as a great solution towards neurodegenerative diseases in general, and are deemed as neuroprotective agents, after having been tested on cells wherein they displayed promising results. Using the MTT method, Aβ_25–35_ treated SH-SY5Y cells’ viability decreased when they were pretreated with the shrimp extracts. The reduction of cell viability was significantly lower in a dose-dependent manner, due to the protective behavior of the shrimp extract. Cells treated with Aβ_25–35_ showed an increased ROS fluorescence in comparison to pretreated cells that showed 23% less fluorescence intensities. Pre-treating the cells with the shrimp extract also demonstrated their protective behavior against TNF-α mRNA, which was previously enhanced by Aβ_25–35_. In parallel, the shrimp extract protected the nerve growth factor (NGF) and brain-derived neurotrophic factor (BDNF) mRNA expression against the action of Aβ_25–35_, but not the BNDF protein expression. Aβ_25–35_ also affected the tropomyosin receptor kinase A (TrkA) protein expression, while at the same time pre-treated cells seemed to function in the opposite way. On the other hand, a downside was noticed in the unexpected increase of the tropomyosin receptor kinase B (TrkB) expression in the pre-treated cells, while the Bax:Bcl-2 ratio, which was supposed to face an increase by the influence of Aβ_25–35_, was stable. However, the effect of Aβ_25–35_ on Caspase-3 seemed to be supported by the extract’s activity. Overall, the shrimp extract partially decreased the ROS production, confirming its protective role towards neurons and against harmful biomolecules, such as Aβ_25–35_, that are linked to neurodegenerative diseases like Alzheimer’s. It must also be noted that the shrimp extract decreased the GSH content, indicating that it can act as a balancing factor between oxidative stress and antioxidants. The extract activated the TNF-α gene, which can function as a pro-apoptotic and anti-inflammatory factor in the JNK pathway. Results suggest that shrimp extracts can prevent SH-SY5Y cells from neurotoxicity and are most likely correlated to the shrimp’s high OA content. Therefore, it was proved that shrimp FA extracts act as neuroprotective agents against inflammation and oxidation, and as protective factors against neurodegenerative disorders [139].

An in vivo study on rats with Aβ-induced memory impairment was conducted to evaluate the neuroprotective effects of liposome-encapsulated ethanolic extracts from shrimp shells, on the rat’s brain. To evaluate the results, the Morris water maze test was conducted along with the object recognition test and a Western blot analysis, in order to examine the brain tissues and protein pathways. The rats were treated with the extracts in pure form or loaded on liposomes for 8 weeks at a dose of 100 mg/kg. No significant difference was noticed after the Morris water maze test between the rats treated with the non-encapsulated extract and the Aβ-treated rats; however, for the rats treated with the encapsulated form, a reduction in the escape time was noticed. This observation indicates that shrimp extracts may impair spatial memory deficits and memory loss in rats with Alzheimer’s disease.

For the purpose of better supporting this theory, the object recognition test was also used. During the training session, there was no specific preference, meaning no difference was observed in the preference index (PI) between the two groups on similar objects, yet when one object was replaced with a novel one, Aβ-treated rats displayed a decrease in the PI, which signified memory and logic dysfunction compared to the encapsulated extract-treated rats which exhibited an increase of PI for the novel object. The obtained outcomes prove that the encapsulated form of ethanolic shrimp extracts may improve cognitive impairments. Additionally, Western blot analysis was used to examine the difference between the two groups of rats on BDNF and its receptor TrkB, which are linked to synaptic plasticity and memory regulation. Rats treated with Aβ_1–42_ displayed a decrease in both Aβ_1–42_ and TrkB expression in the cortex and hippocampus, while rats treated with the encapsulated extract displayed the opposite effect. However, rats treated with the non-encapsulated form exhibited no difference compared to the Aβ_1–42_ group. Growth-associated protein 43 (GAP-43) and postsynaptic density 95 (PSD-95) proteins were tested in a similar way and comparable results were acquired, which confirmed that encapsulated shrimp extracts can attenuate the synaptic plasticity impairment caused by Aβ_1–42_. The neuroprotective behavior of encapsulated shrimp extracts was verified by examining the expression of the extracellular signal-regulated kinase (Erk), the phosphorylation of Erk (p-Erk), and the activation of the Erk signaling pathway. As expected, the expression of p-Erk and Erk displayed an increase in rats treated with the encapsulated shrimp extract, in contrast to those treated with Aβ_1–42_ [140].

Astaxanthin can also be used to prevent cognitive impairment and neurodegeneration in an encapsulated version, due to its non-polar structure that simplifies the process of losing its biological activity. Astaxanthin was tested on an animal model using rats so as to evaluate its effects on neurodegeneration enhanced by the Aβ protein. Astaxanthin did not show any difference in the rats’ spontaneous behaviors, but after being tested on their memory (Morris’s water maze test), rats treated with Aβ_1–42_ took longer to figure out the exit, compared to rats treated with astaxanthin and astaxanthin powder, which exhibited significantly decreased exit time values and increased target time. Rats treated with astaxanthin also had better object recognition, hence the importance of astaxanthin towards the brain’s well-function, as well as its role against memory dysfunction and Alzheimer’s effects, was confirmed. Rats treated with astaxanthin extracted from shrimp shells also demonstrated lower malondialdehyde (MDA) and protein carbonyl levels, and increased inhibition of superoxide anion and glutathione peroxidase (GPx) levels, findings that further support its effect on the decreased oxidative stress in the brain, compared to the control group or the group that was treated with vitamin E. Astaxanthin extracted from shrimp did not show any difference to commercial astaxanthin, a beneficial outcome that highly supports the circular economy pattern since natural astaxanthin can reduce economic expenses while providing the same effects.

Specific regions in the brain, such as CA1 and CA3, spotted in the hippocampus and cortex regions, respectively, are connected to memory and neuronal loss due to the increased Aβ_1–42_ that enhances the effects of Alzheimer’s disease. These regions were evaluated with a view to test the effects of astaxanthin on neuronal survival through Cresyl violet staining. Control groups treated with nothing demonstrated a round and pale-stained nuclei in these specific regions; however, mice treated with Aβ_1–42_ exhibited serious cell death. Indeed, astaxanthin in any from was claimed to exhibit beneficial effects. Commercial astaxanthin and astaxanthin powder (10 mg/kg) initiated a decrease in the neuronal degeneration in these brain regions, while the astaxanthin extract showed on the contrary a decrease in neuronal degeneration only in the cortex region. Rats treated with astaxanthin for 30 days in all forms also displayed a reduction in the positive staining of the Aβ that surfaced on the rats treated with Aβ_1–42_. Therefore, the extract from *Litopenaeus vannamei* was considered beneficial for the prevention of neuronal degeneration and memory loss in the protection of neurons against the negative impact of Alzheimer’s [141]. Consequently, the UFAs and astaxanthin of shrimp had a rather significant beneficial action on the memory and synaptic plasticity impairments, which further supports the need for regular consumption of shrimp in order to fully reap its neuroprotective benefits against Alzheimer’s disease.

### 4.5. Shrimp Extracts Effects on the Kidney

Kidneys are important organs responsible for filtering the blood and ensuring it is clean and healthy before its distribution in the whole body. Various important hormones are also produced in the kidney, such as erythropoietin (EPO), which is accountable for the production of red blood cells in the bone marrow. Kidney diseases and kidney failure are widespread diseases induced by a plethora of factors, mostly free radicals, and are also very common among people suffering with diabetes or groups that overconsume other substances, like alcohol, that are able to cause oxidative stress [142].

Astaxanthin extracted from shrimp waste displayed beneficial and nephroprotective effects on diabetic rats. Rats with diabetes showcased a decrease of GSH levels, catalase, and superoxide dismutase in the kidneys, with percentages of 26%, 36%, and 18%, respectively compared to the control group. Diabetic rats treated with astaxanthin deriving from the shrimp extract, on the other hand, displayed an increase of the GSH levels, catalase, and superoxide dismutase in the kidneys, with percentage of 19%, 35%, and 17%, respectively. GPx levels also increased in the diabetic rats’ kidneys treated with astaxanthin (78 ± 4 nkat/mg protein), compared to diabetic rats without (39 ± 3 nkat/mg protein). Lastly, protein oxidation products were significantly lower in the diabetic rats treated with astaxanthin from the shrimp extract in comparison to diabetic rats. Histological examination was also applied in order to evaluate the cell structure of the kidneys in diabetic rats treated with the shrimp extract, where normal cell construction was noticed. On that account, astaxanthin extracted from shrimp shells indicated significant capability of suppressing the oxidative stress caused by diabetes on kidney function. Oxidative damage of the protein and lipid peroxidation of the membrane and lipoproteins were also inhibited after the astaxanthin treatment [143]. Therefore, shrimp extracts are beneficial towards insulin resistance and diabetes, and can also be preventative agents of nephropathy caused by the hyperglycemia, which is linked to diabetes.

### 4.6. Shrimp Extracts on Skin Benefits

Maintaining the health of human skin constitutes a significant and critical research field. In general, our skin is composed of the epidermis, dermis, and hypodermis, and its main function is to protect the interior environment from exterior stimuli, namely potentially harmful agents that may come in contact with the skin (viruses, sunlight, bacteria, microbes, etc.), or changes in the weather that would affect the homoeostasis of the body. It also keeps the balance between many substances and the rest of the body and is also linked to our metabolism and immune response [144].

Astaxanthin, due to its anti-inflammatory and antioxidant properties, can possibly be used in the cosmetic field so as to provide its natural properties against conventionally used synthetic chemicals [145]. Astaxanthin extracted from *L. vannamei* was tested on human dermal fibroblast cells and the recovered results were very promising. Singlet oxygen scavenging was used to determine the non-radical ROS affiliated with many skin disorders. Indeed, the extract inhibited strong singlet oxygen quenching activity with an EC_50_ value of 9.2 ± 0.5 μg/mL, which indicated its potential use as a natural antioxidant for the skin and as a preventative agent against future diseases. Tyrosinase activity was also tested, as it is linked to the control of melanin production. After the cells’ treatment with astaxanthin, the shrimp extracts inhibited IC_50_ value against tyrosinase at a more effective level (12.2 ± 1.5 μg/mL) than all other extracts tested.

Human dermal fibroblast cells WS1 were chosen in order to evaluate the toxicity of astaxanthin; however, results after 24 h were not any different than the control group and the cell viability was found up to 90%, suggesting that astaxanthin is not toxic for human skin and could be used in future cosmetic products. Induced cytotoxicity from astaxanthin was also tested, and the morphology did not change after treatment, proving once again the safety of astaxanthin on human cells [73]. In essence, astaxanthin was confirmed as a safe substance for the human skin and as a strong antioxidant, a very important outcome considering the growing global pollution phenomenon the human skin must endure, with astaxanthin being effective against all pollutants.

### 4.7. Shrimp Extracts on Immune Responses

The immune system can be considered as the primary defense mechanism of the human body against pathogens (bacteria, viruses, germs, etc.); by the time its harmony is disrupted through a series of chain reactions, a living organism may suffer tremendously or even die. Through fast cooperation of special receptors and antibodies each created for a specific reason, a whole defense mechanism is built known as our immune system [146].

Helicobacter pylori is a very common bacteria that infects more than 50% of the world’s population. Its symptoms may vary from chronic gastritis and peptic ulceration to gastric adenocarcinoma and primary gastric lymphoma [147]. However, chronic inflammation of the gastrointestinal system may enhance other diseases, depending on the immune system of the host. Helicobacter pylori is mainly responsible for spreading T-helper 1 (Th1) and T-helper 2 (Th2) cell responses [148]. Th1 and Th2 subsets display definite patterns of cytokine secretion, with the Th1 phenotype distinguished by a predominance of interferon-gamma (IFN-γ), which contributes to the persistence of inflammation and the inhibition of a potent Th2 response. More specifically, increased levels of IFN-γ establish a Th1-dominant microenvironment, inhibiting interleukin 2 (IL-2) activity, which plays an essential role in the priming of Th2 responses and the protection against this infection [149]. Many antioxidants have captured attention as they are able to slow down the Th1-type activation pathways in order to stimulate the production of Th1 and Th2 cytokines [150].

Astaxanthin has also gained interest as a natural beneficial antioxidant, and extracts from *H. pluvialis* shrimp were used on H. pylori-infected mice so as to test cytokine release caused by astaxanthin and its potent role against the spreading. Cells from H. pylori-infected mice did not produce IFN-γ until four weeks after the initial consumption of astaxanthin. However, the values implied a slight trend towards the upregulation of IFN-γ, with the highest concentration of astaxanthin (40 mg) being recorded after four weeks of treatment. Nevertheless, after six weeks of treatment, splenocytes produced significantly elevated levels of IFN-γ in H. pylori-infected mice. Moreover, the levels of IL-10 in splenocytes were tested to evaluate the cytokine profile of the *H. pylori*-inoculated mice and a significant production of IL-10 was produced after six weeks of treatment in comparison to the infected mice, indicating that astaxanthin can affect the mediating of inflammation caused by *H. pylori* bacteria. The synthesis of IL-2 was increased in splenocytes of infected mice treated with 10 mg of astaxanthin, but after six weeks, the levels of IL-2 were notably bigger in mice treated with 40 mg of astaxanthin than those of mice supplemented with 10 mg astaxanthin or untreated mice. Hence, large amounts of astaxanthin can be harmful, but smaller ones are confirmed as beneficial towards the well-being of our immune system [151].

Therefore, regulated amounts of astaxanthin can be used to control the cytokine profile during *H. pylori* infection, and further in vivo studies in humans could be revolutionary for the treatment of this very common bacteria genus.

### 4.8. Shrimp Extracts on Dementia

Dementia is a disease/syndrome distinguished by memory, language loss, and many other symptoms such as lack of clarity of thoughts and an incapability to attend to basic life needs. Dementia can be induced by the Alzheimer’s disease as well [152]. Several factors can lead to dementia that may initiate from many different body parts other than the brain. Heart arrhythmias, a relatively simple health problem, could form a clot that migrates to the brain, disrupting the blood flow and immediately affecting the brain tissue and its functions, concluding in early stages of dementia or worse conditions. Suffering from more than one common disease is also proved to increase the chances of developing dementia due to the pressure exerted on the organs, which finally reaches the brain [153].

The brain consists of many n-3 FAs such as DHA, which is sourced from seafood and can be metabolized by EPA and ALA. As predicted, n-3 FAs are very important for the proper function of the brain, including the prevention of dementia. In this context, an in vivo study was conducted to analyze the n-3 FAs from various marine animals, including the effects of shrimp on the apolipoprotein E, type ε4, allele (APOE-ε4), a gene encoding the lipid protein responsible for intercellular trafficking of cholesterol and other lipids involved in the brain’s structure, which is also highly tethered to dementia. There were many participants of different sexes, habits, ages, etc., who volunteered for this research and were treated with n-3 FAs for 5 years. The overall characteristics and traits of every person played an important role in the results. People treated with one or more seafood meals per week did not reveal a decline in global cognition; they did, however, demonstrate slower rates of decline in semantic memory and perceptual speed compared to others who consumed less seafood, and therefore less n-3 FAs. A total of 178 APOE ε4 carriers were discovered in total among volunteers, while seafood consumption exhibited a slower decrease in the global cognitive score, episodic memory, semantic memory, and perceptual speed in this five-year span. Larger traced amounts of n-3 FAs indicated slower declines in the semantic memory and perceptual speed factors, but not on the global cognition, episodic memory, and working memory, a fact that implied that larger amounts than necessary could harm rather than boost the brain’s performance. People with covariates for stroke, diabetes, hypertension, etc., displayed reduced effects of seafood on global cognitive decline and episodic memory, but not on semantic memory and perceptual speed. Moreover, long-chain n-3 FAs seemed to be 32% less effective on working memory, while the rest of the factors were also altered but not significantly [154].

In a summary, there are signs that n-3 FAs possess a neuroprotective role against Parkinson’s disease; nonetheless, more trials are required in order to fully comprehend their potency against Parkinson’s and neurodegenerative diseases, in general.

**Table 6:** Studies, interventions and clinical trials on the benefits of bioactive lipid extracts derived from shrimp against chronic disorders including cancer, diabetes, retinopathy, Alzheimer’s disease, kidney disease, other neurological disorders, and depression.

Hypothesis/Intervention	Study Design/Parameters Examined	Main/Concluding Observed Benefit(s)	Specific Benefits/Other Benefits/Mechanism of Action	Year of Study	References
This study aimed to characterize antiproliferative fractions from farmed shrimp *Litopenaeus vannamei*.	Shrimp muscle extracts were tested for their antiproliferative effects using the MTT bioassay.	M.11.B.1 sub-fraction, in addition to EPA, demonstrated antiproliferative effects on a prostate cancer cell line.	A GI50 = 58.3 ± 10 μg/mL EPA value indicated the contribution of this compound to M.11.B bioactivity. Sub-fraction M.11.B.1 showed the highest antiproliferative activity (Table 3), with a GI50 = 26.6 ± 8 μg/mL.	2021	[124]
This study aimed to explore the antitumoral activities of the lipophilic constituents of shellfish species.	Cytotoxicity potential was determined against five different human cancer cells.	The Argentine red shrimp lipid extract was the most cytotoxic lipid among all investigated cancer cells.	-	2021	[72]
This study aimed to evaluate *L. vannamei* muscle lipid fractions on cancer cell lines and to identify their chemical components.	Muscle extracts were tested on their cancer cell viability and morphology using MTT and fluorescence microscopy.	White shrimp muscle extracts show antiproliferative effects on human cancer cell lines, especially sub-fractions C5-3 and C5-4 containing β-carotene, EPA, cholesterol, and phthalate.	Lipid subfraction C5-4 demonstrated potent antiproliferative effects on breast adenocarcinoma (MDA-MB-231), inducing morphological changes indicative of apoptosis.	2022	[125]
This study explored the combined effects of astaxanthin (from shrimp), β-carotene, and lutein on molecular events induced in MCF-7 cells.	MCF-7 cells treated with shrimp-derived astaxanthin, β-carotene, and lutein were analyzed for their cellular uptake, cytotoxicity, and apoptosis.	Shrimp-derived astaxanthin potentiated anti-proliferation of MCF-7 cells when co-treated with other carotenoids of green leafy origin, like β-carotene and lutein.	Shrimp-derived astaxanthin, along with β-carotene and lutein, was found to synergistically induce apoptosis through modulation of cyclin D1, p53, Bax, and Bcl-2 expressions by arresting cell cycle at G0/G1 phase.	2017	[126]
This study aims to determine the anticancer cell proliferation activity in vitro.	The antiproliferative activity was determined towards three different human lung cancer cell lines (H1975, H3255, and H441) by MTT assay.	The proliferation assays show that the extract had a high potency to inhibit the H1975 cancer cells.	-	2020	[127]
This study aimed to investigate how shrimp oil affects glucose regulation in obese rats.	Male CD rats on high-fat and fructose diet were divided accordingly: control (0% shrimp oil), SO10 (10%), SO15 (15%), SO20 (20%) shrimp oil, replacing lard for 10 weeks.	Shrimp oil is a promising ingredient for the prevention and treatment of insulin resistance and T2DM.	Shrimp oil improved (*p* < 0.05) oral glucose tolerance, insulin response, and homeostatic model assessment-estimated insulin resistance index, decreased serum insulin, leptin, HbA1c, and free FAs, while it also increased adiponectin.	2017	[133]
The effects of fermented shrimp shells (SS) on modulating hepatic enzymes for glucose metabolism and attenuating lipid peroxidation were illustrated.	The effect of fermented SS was studied on insulin resistance in FL83B hepatocytes and diabetic rats.	In vitro and in vivo experiments confirmed that fermented SS possess protective effects against diabetic complications like hyperglycemia, hyperlipidemia, lipid peroxidation, and kidney dysfunction.	Daily oral supplement of fermented SSs to streptozotocin/nicotinamide (STZ/NA)-induced diabetic rats for 7 weeks reduced plasma glucose and insulin resistance. It enhanced hepatic glucose catabolism by increasing hexokinase and glucose-6-phosphate dehydrogenase activity, while reducing the glucose-6-phosphatase activity. Fermented SSs also lowered plasma total cholesterol (TG), triacylglycerols, LDL cholesterol, and liver TG, as well as lipid peroxidation in diabetic rats.	2022	[134]
This study was held so as to assess whether the LCn-3 PUFAs intake correlates with reduced sight-threatening diabetic retinopathy incidence in T2DM patients over the age of 55 years old.	A total of 3614 patients at the age of 55 to 80 years old with T2DM had their dietary intake assessed yearly via a validated questionnaire during the PREDIMED study.	All findings, which were consistent with the current model of the pathogenesis of diabetic retinopathy and data from experimental models, declared fish-derived LCn-3PUFAs as healthy fats.	Meeting the target of at least 500 mg/day of dietary LCn-3PUFAs is associated with a reduced incidence of severe diabetic retinopathy in individuals older than 55 years with T2DM.	2016	[21]
In this study, the effects of shrimp extract (SE) and shrimp oil (SO) on fat accumulation, adipogenesis, and lipogenesis in 3T3-L1 adipocytes were investigated.	Fully differentiated 3T3-L1 preadipocytes were stained with “Oil Red O” to study the fat accumulation and total RNA extraction performed, in order to measure the overall gene expression.	SO extracted from shrimp processing by-products reduced fat accumulation in 3T3-L1 cells.	SO showed no significant effect on Scd1 and FASN, as well as in the mRNA expression of Glut-4.	2021	[19]
This work aimed to estimate indications of anti-obesity and hypolipidemic effects of the extracts obtained from pink shrimp residue.	Anti-obesity and hypolipidemic effects in Swiss Mus musculus treated with shrimp extracts on a 30-day high-fat diet, were evaluated.	Related to the anti-obesity and hypolipidemic effects, the extracts showed good activity potential, especially the supercritical extract when applied at a concentration of 50–100 mg/kg per d.	Normal mice responded with hypolipidemic effects to supercritical fluid extraction (SFE) extract of 100 mg/kg per day, while all concentrations of Soxhlet extraction (SOX) and SFE extracts, reduced cholesterol and triacylglycerols in LDL receptor knockout mice, compared to the control group.	2015	[122]
This study aimed to assess if the acetone 4-2A extract from shrimp by-products protects neurons from Aβ-induced neurotoxicity through neurotrophic, anti-inflammatory, and antioxidative mechanisms.	The study examined 4-2A’s antioxidative and anti-inflammatory effects on Aβ_25–35_-treated SH-SY5Y cells using MTT and lactate dehydrogenase (LDH) assays, quantitative PCR (qPCR), and Western blotting.	4-2A effectively protected SH-SY5Y cells against Aβ-induced neuronal apoptosis/death by suppressing inflammation and oxidative stress and up-regulating NGF and TrkA expression.	An amount of 20 μM of Aβ_25–35_ reduced SH-SY5Y cell viability, NGF, TrkA, and GSH levels, while increasing ROS, TNF-α, BDNF, TrkB, Bax/Bcl-2 ratio, and Caspase-3. Treatment with 4-2A attenuated most changes.	2017	[139]
This study aimed to investigate the neuroprotective effect of ethanolic extract from shrimp shells (EESS) by using liposome encapsulation.	Ethanolic extracts from white shrimp shells and ethanolic extract-loaded liposomes for neuroprotection in Aβ_1–42_-induced memory impairment in rats, with commercial astaxanthin as control, were tested.	The present study provided evidence that the neuroprotective property of the EESS-loaded liposome could be a promising strategy for Alzheimer’s disease (AD) protection.	The proposed activity of the EESS-loaded liposome was through promoting the expression of learning and memory-related proteins BDNF/TrkB, as well as synaptic proteins GAP-43 and PSD-95 in the cortex and hippocampus via p-Erk1/2/Erk1/2.	2022	[140]
This study aimed to investigate the effects of astaxanthin extract and encapsulated astaxanthin on cognitive impairment and neurodegeneration in an animal model of Aβ-induced AD.	Rats divided into eight groups received treatments orally for 30 days, followed by behavioral tests and hippocampus/cortex analysis.	Astaxanthin from shrimp (*Litopenaeus vannamei*) shells protected Wistar rats from Aβ_1-42_-induced oxidative stress and resulted in improved learning and memory.	The Aβ_1-42_ infused group exhibited cognitive decline and increased memory loss in behavioral tests, alongside elevated oxidative markers in brain regions. Astaxanthin powder (10 mg/kg) outperformed both the astaxanthin extract and vitamin E.	2019	[141]
This study investigated the hypoglycemic and antioxidant effects of shrimp astaxanthin on the kidneys of alloxan-induced diabetic rats.	Six rats per group: control (C), diabetic (D), diabetic + Astaxanthin (D + As) in olive oil, and diabetic + olive oil (D + OO). Antidiabetic effects were assessed both in plasma and kidney tissue.	Astaxanthin extracted from the shell waste of shrimp had a significant hypoglycemic effect on alloxan-induced diabetic rats.	Astaxanthin can be effective in inhibiting hyperglycemia, oxidative stress, and cell damage in kidneys, by enhancing antioxidant enzyme activity, scavenging ROS, and eventually by contributing to the improvement of tissue dysfunction in diabetic rats.	2015	[143]
This study aimed to evaluate astaxanthin levels in shrimp shells for antioxidant and anti-tyrosinase activities, and also to assess its cytotoxic effects on human dermal fibroblasts for skin health use.	Antioxidant activities were assessed using DPPH, ABTS radical scavenging, β-carotene bleaching, and singlet oxygen quenching assays.	Findings confirmed that shrimp astaxanthin can be used as a functional ingredient in skin health products, ensuring both the efficacy and safety.	Astaxanthin demonstrated strong antioxidant and tyrosinase inhibition without cytotoxicity to human dermal fibroblasts. Optimal activity was observed at concentrations of 10–20 µg/mL.	2019	[73]
In this study, the effects of varying oral doses of astaxanthin from shrimp cephalothorax on cytokine release in H. pylori-infected mice splenocytes were examined.	6-8-week-old female mice were divided into three groups (*n* = 20 each) receiving daily oral doses of 10 or 40 mg astaxanthin for six weeks.	The results of the present study suggest that the administration of astaxanthin from shrimp cephalothorax may modulate the cytokine profile during H. pylori infection.	After six weeks, a trend towards IFN-γ upregulation was found (40 mg; *p* < 0.05) and a significant dose-dependent increase of IL-2 and IL-10 (both *p* < 0.05) was observed.	2019	[151]
This study was held in order to examine the association between the consumption of seafood and long-chain n-3 FAs with the change in five cognitive domains over an average of 4.9 years.	We analyzed 915 participants (mean age 81.4 ± 7.2 years, 25% men) with cognitive assessments and dietary data deriving from a food frequency questionnaire.	These results suggested protective relations of one meal per week of seafood and long-chain n-3 FAs against the decline in multiple cognitive domains.	Seafood consumption correlated with a slower decline in semantic memory (*b* = 0.024; *p* = 0.03) and perceptual speed (*b* = 0.020; *p* = 0.05) was observed. Also, higher intake levels of ALA, were associated with slower global cognitive decline, only in APOE-ε4 carriers.	2016	[154]

## 5. Methods

The bibliography was gathered through searches on Scopus/Science direct and PubMed using the following keywords: shrimps and lipid, composition, content, lipid content, lipid composition, phospholipid, phospholipid content, phosphatidylcholine, phosphatidylethanolamine, fatty acid, fatty acid profile, fatty acid content, fatty acid composition, n-3, n-6, unsaturated fatty acids, UFA, polyunsaturated fatty acids, PUFA, DHA, EPA, docosahexaenoic acid, eicosapentaenoic acid, ALA, α-linoleic acid, carotenoid, astaxanthin, anti-inflammatory, antithrombotic, antioxidant, anti-atherogenic, cardio-protective, cardio-protection, cardiovascular health, inflammation, thrombosis, oxidative stress, atherosclerosis, cardiovascular diseases, hypercholesterolemic, anti-hypercholesterolemic, anti-dyslipidemia, dyslipidemia, antilipidemic, neuroprotective, neurodegenerative disorders, Alzheimer, Parkinson, dementia, antidiabetic, diabetes, metabolic syndrome, anti-glycemic, renal, kidney, and glomerulosclerosis. These keywords were combined to obtain the desired results of the searched articles.

An extensive search was conducted on shrimp species including *Litopenaeus vannamei*, *Penaeus Kerathurus*, *Aristaeomorpha foliacea*, and *Parapenaeus longirostris*; however, only the first species displayed a sufficient number of studies, demonstrating the research gap on the remaining Mediterranean shrimp. A time frame was used from 2015 and after for our search, so as to include new and upcoming results and information on shrimp and their use in marine drugs. However, due to the research gap, especially in the lipid composition of shrimp, the time frame of the searched articles was extended in order to find more information for this subject. From the total number of articles that were studied, a lot of them were excluded from this review, for various reasons (some of them were deleted or duplicates). In order to include an article in our review, we first evaluated the titles, then the abstracts, and lastly the keywords. Papers were excluded if they failed to satisfy the English language writing standard, if they were deemed to not be research articles, or they were not within the time limit set. For the theoretical part of this review, we consulted articles and reviews, within the time limit set from 2015 up to the date of publication.

## 6. Conclusions

This review provides an in-depth analysis of the nutritional worth and bioactive characteristics of shrimp and their by-products, emphasizing on the noteworthy anti-inflammatory, antioxidant, antithrombotic, antitumor, antidiabetic, and other properties of these consumables. Shrimp are rich in bioactives, including n-3 PUFAs, PLs, carotenoids, and phenolics, and have been demonstrated in numerous in vitro, in vivo, and randomized controlled trials to have positive effects on human health, using a variety of mechanisms that reduce inflammation. Subsequently, this manuscript describes the various applications of shrimp lipids and lipophilic bioactives in several industries, including food (as fish feed additives in aquaculture or natural flavor additives), pharmaceutical (as additives in drug formulations), nutraceutical (as n-3 supplementary diets), and cosmeceutical (as skin care products), highlighting their significant roles in humans and other organisms on managing chronic diseases associated with inflammation. Scientific research on shrimp concludes that when they are included in a healthy, balanced diet, they act as great health-promoting and health-protective factors.

This review also examines the financial and environmental benefits of recycling shrimp by-products, particularly the cost-effectiveness of utilizing all shrimp parts and avoiding the costly and harmful to environment waste management, which is in line with the ideas of circular economy and sustainability. Notably, the ability to gather these species through aquaculture contributes to a more environmentally friendly production of these bioactives and strengthens the circular economy. Shrimp by-products include a wealth of nutrients and bioactive chemicals, making them an appealing product for cosmetics and other sectors; however, more research is needed to determine both how to extract them efficiently and what other hidden applications they might possess. Finally, this review highlights the need for further research on shrimp lipid bioactives such as carotenoids, phenolics, sterols, and PLs, which are believed to display significant impact on our cardiovascular system.

## Figures and Tables

**Table 2:** Composition of neutral lipids in meat/flesh and by-products/waste extracts of different shrimp species.

	Meat/Flesh			By-Products/Waste		
	*L. vannamei*	*M. kerathurus*	*P. monodon*	*L. vannamei*	*M. kerathurus*	*P. borealis*
Neutral Lipids (% of TLs)	-	22.5–22.7	-	-	51.1–51.5	-
MAGs (% of TLs)	0.34	-	0.32–0.40	1.86	-	-
DAGs (% of TLs)	0.43–2.2	-	0.53–0.59	0.80	-	-
TAGs (% of TLs)	1–1.28	7.9–8.1	2.27–2.69	2.23	22.93–22.96	8.31–19.01
Free FAs (% of TLs)	3.19	0.6–0.8	3.81–4.35	14.55–18.14	0.5–0.7	0.0–0.80
Waxes (% of TLs)	-	0.1	-	-	-	0.58–0.76
References	[36,55]	[42]	[36]	[36,50]	[42]	[19]

**Table 3:** Composition of polar lipids in meat/flesh and by-products/waste extracts of different shrimp species.

	Meat/Flesh		By-Products/Waste	
	*L. vannamei*	*P. monodon*	*L. vannamei*	*P. borealis*
Polar lipids (% of TLs)	77.02–77.56	-	67.09–68.71	-
Phospholipids (% of PLs)	-	-	47.10–51.21	59.52–68.88
Glycerophospholipids (% of TLs)	77.29	74.40–75.44	67.90	-
PtdCho (% of PLs)	52.8	-	-	-
PtdEtn (% of PLs)	24.7	-	-	-
PtdIns (% of PLs)	1.1	-	-	-
References	[36,55]	[36]	[36,50]	[19]

**Table 4:** Composition of marine-derived bioactives in meat/flesh and by-products/waste extracts of different shrimp species.

		Astaxanthin (μg/g)	β-carotene (μg/g)	α-tocopherol (μg/g) FW	Sterol Esters % of TLs	Sterols mg/100 g FW	Cholesterol mg/100 g FW	Total Polar Compounds (mg GAE/g)	References
Meat/flesh	*L. vannamei*	0.69–0.77	0.05–0.13	16.7	-	-	34.94	-	[23,71]
	*M. kerathurus*	-	-	-	0.2–0.4	146.78–173.02	130.21–158.37	-	[42]
	*P. monodon*	-	-	21.7	-	-	40.91	-	[23]
	*P. muelleri*	-	-	16.4	-	118.13–126.27	41.64	-	[23,72]
	*P. borealis*	-	-	-	-	-	-	0.19	[63]
By-products/waste	*P. serratus*	-	-	-	-	-	-	6.0–11.1	[62]
	*P. varians*	-	-	-	-	-	-	4.4–10.5	[62]
	*L. vannamei*	19.20	-	34.1	-	-	50.47	-	[23,73]
	*M. kerathurus*	-	-	-	1.9–2.3	520.9–539.9	511.81–531.07	-	[23,42]
	*P. monodon*	2.91	-	35.3	-	-	55.93	-	[23,25]
	*P. japonicus*	5.8	-	-	-	-	-	-	[25]
	*F. chinensis*	7.1	-	-	-	-	-	-	[25]
	*P. borealis*	-	-	-	-	-	-	0.15–0.28	[19,63]
	*C. crangon*	1.8–2.9	-	-	-	-	-	-	[53]
	*M. rosenbergii*	15.68	-	-	-	-	-	-	[25]

## Data Availability

Not applicable.
